# Realist Approach to Social Policies (RASP) study to reduce socioeconomic health inequalities through systems change: protocol for a research project combining mixed-methods realist research with institutional action research

**DOI:** 10.1136/bmjopen-2024-088571

**Published:** 2024-06-13

**Authors:** Jantien Van Berkel, Ernst-Jan de Bruijn, Maikel Waardenburg, Yvonne la Grouw, Eline van Bennekom, Hilje van der Horst, Susanne Tonnon, Milou Haggenburg- Mohammed, Annemien Haveman-Nies, Tamara Madern, Marike Knoef, Emely de Vet

**Affiliations:** 1 Interdisciplinary Social Science, Public Health, Utrecht University, Utrecht, The Netherlands; 2 Consumption and Healthy Lifestyles, Wageningen Universiteit en Research, Wageningen, The Netherlands; 3 Institute of Tax Law and Economics | Department of Economics, Leiden University, Leiden, The Netherlands; 4 Law, Economics and Governance, School of Governance, Utrecht University, Utrecht, The Netherlands; 5 Debt and Collection, Hogeschool Utrecht, Utrecht, The Netherlands; 6 Onderzoek en Statistiek, Gemeente Amsterdam, Amsterdam, The Netherlands; 7 AGORA Academische Werkplaats, GGD Noord en Oost Gelderland, Warnsveld, The Netherlands; 8 Tilburg School of Economics and Management, Tilburg University, Tilburg, The Netherlands; 9 Tilburg School of Humanities and Digital Sciences, Liberal Arts and Sciences, Tilburg University, Tilburg, The Netherlands

**Keywords:** Health Equity, Community-Based Participatory Research, MENTAL HEALTH, Overweight, Smoking Reduction

## Abstract

**Introduction:**

Health inequalities are rooted in inequality in vital resources for health, including financial resources, a supportive informal network, a stable living situation, work or daytime activities or education and literacy. About 25% of Dutch citizens experience deprivation of such resources. Social policy consists of crucial instruments for improving resources in those groups but can also have adverse effects and lead to additional burdens. This project aims to contribute to the reduction of health inequalities through (1) a better understanding of how social policy interventions can contribute to reducing health inequality through the redistribution of burdens and resources and (2) developing anticipatory governance strategies to implement those insights, contributing to a change in social policy systems.

**Methods and analysis:**

Two systems approaches are combined for establishing a systems change in the Netherlands. First, a realist approach enables insights into what in social policy interventions may impact health outcomes, for whom and under what circumstances. Second, an institutional approach enables scaling up these insights, by acknowledging the crucial role of institutional actors for accomplishing a systems change. Together with stakeholders, we perform a realist review of the literature and identify existing promising social policy interventions. Next, we execute mixed-methods realist evaluations of selected social policy interventions in seven municipalities, ranging from small, mid-size to large, and in both urban and rural settings. Simultaneously, through action research with (national) institutional actors, we facilitate development of anticipatory governance strategies.

**Ethics and dissemination:**

This study is not liable to the Medical Research Involving Subjects Act (WMO). Informed consent to participate in the study is obtained from participants for the use of all forms of personally identifiable data. Dissemination will be codeveloped with target populations and includes communication materials for citizens, education materials for students, workshops, infographics and decision tools for policy-makers and publications for professionals.

**STRENGTHS AND LIMITATIONS OF THIS STUDY**

- The cross-domain systems approach expands understanding beyond traditional health systems by integrating social policy as potential systems for health.
- Realist evaluation provides insight into how social policy contributes to health outcomes and prepares for strategic uptake and upscaling.
- Diverse settings are represented, including both urban and rural municipalities, capturing variations in vulnerability and social policy practices.
- The ‘health imperialism’ critique suggests that evaluating policies primarily focused on resource distribution may raise ethical concerns about the prioritising of health outcomes; however, recognising the bidirectional interplay between resource allocation and health outcomes is crucial.
- Evolving political contexts may present challenges in sustaining support for social policy initiatives due to changing political climates.

## Introduction

Health inequalities are rooted in social structures and systems. Approximately 25% of Dutch citizens find themselves in vulnerable positions,^1^ meaning that they lack a combination of financial resources, a supportive informal network, a stable living situation, work or daytime activities, education and literacy. Such vulnerabilities can be understood as deprivation in different forms of resources, including economic, social, cultural and personal resources.^1^ While deprivation may exist in a sole domain, it tends to cumulate across domains.^2^ All such forms of resources separately have been shown to be related to health outcomes, including years in good health,^3^ but especially when deprivations cumulate, health loss may be substantial.^4^ Health loss through adverse lifestyle behaviours (such as problematic alcohol use, smoking and energy-balance-related behaviours) and impaired mental health among individuals in vulnerable positions have been prioritised to reduce health inequalities.^5^ However, directly targeting mental health and lifestyle behaviours in vulnerable groups has been proven to be problematic, especially at the population level, when the underlying deprivations in resources are not addressed.^2^ Resources, such as income and social networks, are thus essential conditions for better mental health and lifestyle behaviours and, relatedly, the reduction of health inequalities.

In welfare states, social policies aim to redistribute resources among citizens, and thereby ameliorate such deprivations of citizens in vulnerable positions. Potentially, they can be tailored towards improving the conditions for health gains in vulnerable groups. However, the impact of social policies, via resources as necessary conditions and mechanisms such as increased agency and reduced stress and burdens, on health outcomes is ill understood. While health benefits are a desired outcome of policies that target vulnerabilities, they are usually not the main target of social policies. Such desired, though unintended, outcomes of policy are best unravelled by understanding social policies as embedded in complex systems with intended and unintended positive and negative outcomes across a range of levels, domains and actors. This means that social policies can sustainably improve but also diminish resources (eg, financial resources, a supportive social network, employment) for vulnerable groups. Improving health behaviour and mental health is essential for people in vulnerable groups to reduce health inequalities.^5^ Improving resources is expected to contribute to those necessary improvements, through reducing stress and improving agency.

With this project, we target two groups of citizens in vulnerable positions that are subject to social policy: (1) recipients of social assistance benefits and (2) people with (a risk of) overindebtedness. In the Netherlands, about 420 000 people receive social assistance (69%>2 years, 41%>5 years) while about 650 000 households face problematic debts.^6 7^ While both groups are primarily defined by deprivation in economic resources, due to the compounding nature, both groups can be expected to also dispose of less other resources. In line with these types of resources being conditions for health status, among both groups, the prevalence of (mental) health problems is relatively high.^8–10^ An extensive summary of the state-of-the-art of research into the effects of social assistance benefits policy and debt policy on the health of their target populations can be found in online supplemental file 1.

10.1136/bmjopen-2024-088571.supp1 Supplementary data

To better understand, and ultimately tailor, the way in which social policies contribute to health outcomes for those two groups, municipalities are a strategic point of entry. In the decentralised system of the Netherlands, the national government determines the modalities of social policies such as social assistance benefits and debt policy while local welfare departments are responsible for processing applications, paying out the monthly benefit, offering employment services, counselling and monitoring claimants, and detecting and sanctioning noncompliance and benefit fraud. There are major differences between municipalities in both the size and composition of the population and causes of vulnerability within the population. In addition, they differ in the available resources for social policy. Therefore, insight is needed into the way in which social policies are embedded in local social policy systems and generate health outcomes.

## Aim and research questions

### Systems change towards social policies for health

With this project, we aim for a systems change in social policies through (1) better understanding of how social policy can contribute to reducing health outcomes through improving the redistribution of resources and (2) implementing those insights to realise conditions necessary to reduce health inequalities. We generate transferable insights that will help the assessment and implementation of social policy interventions that maximise health potential of two vulnerable groups on multiple system levels (ie, national and local government, professionals and citizens). Through those insights coupled with the development of governance strategies that explicate desired futures, we move from a situation of health impacts as desired but unintended outcomes of social policy, towards a situation where municipalities can better adopt health impact as an intended outcome of their social policies (eg, a social policies system for health).

To reach this objective, we will answer the following research questions for the aforementioned two groups (ie, social assistance benefits receivers and people with (a risk of) problematic debt):

1. What in social policy interventions works to generate health outcomes through the redistribution of resources and burdens, for whom and under what circumstances?
2. How can the institutional field (ie, professionals, government) translate insights from RQ1 into strategies to realise conditions to reduce health inequalities?

## Methods and analysis

### Systems approach: realist approach and institutional approach

We combine two system approaches tailored to the distinct objectives of this project. To generate theory-based-empirically tested insights into what in social policy works to generate health outcomes, for whom and under what circumstances, we use a realist approach. First, theory-based insights are developed in a realist review. Based on the initial theory, together with stakeholders, existing potentially impactful social policy intervention are selected. Second, to test these insights empirically, realist evaluations of the selected social policy interventions are conducted. Furthermore, to be able to scale up these insights among institutional actors, we facilitate the development of governance strategies, acknowledging the crucial role of institutional actors to realise a systems change, using an institutional approach.

These two system approaches are linked to the two research questions.

### Realist approach

We adopt a realist approach to social policies for four reasons: (1) the focus on mechanisms; (2) the complexity of social policy interventions and systems; (3) the actor perspective and (4) stakeholder engagement.

### Mechanisms

Previously, conventional evaluations of interventions focused on experimental designs, with the Randomized Controlled Trial as the golden standard. This approach has been criticised for its narrowness of scope,^11–13^ and ‘black boxing’ effectiveness, especially for complex interventions^14^ such as social policy interventions. Alternative approaches have been called for.^12–15^ In this study protocol, we employ such a novel paradigm: the realist approach.

Realist approach is a theory-driven research approach, based on realist philosophy of science.^15 16^ The underlying assumption is outcomes are produced by mechanisms: the causal power of things (ie, material things, but also social structures and relationships) to affect other things in specific ways.^17^ Whether or not the causal power is activated depends on context, for example, circumstances.^15^ The aim is to generate insights into how contexts and mechanisms interact, resulting in outcomes,^16 18^ thereby unravelling the black box of what works for whom and under what circumstances. This resolves the knowledge gap in current literature (see ‘state-of-the-art’); it is not well understood how social policy works (ie, mechanisms), and in what context it works, especially in the Dutch welfare regimen. Moreover, insight in the combination of mechanisms and context ensure transferability of the findings. Transferability is particularly relevant, as it allows to develop strategies to roll out or scale up interventions. In this project, these insights are based on theory (realist review) and then empirically tested (realist evaluations).

### Complexity

Not only can social policy interventions be considered complex interventions, meaning they contain multiple components, the system in which social policy takes place is also complex. For example, other policies might overlap (ie, permeable system boundaries), which means that professionals and citizens are not only exposed to the social policy intervention under study. In addition, social policy interventions are ‘nested’ in other social systems, like national policies on financing structures and legislation, that influence (the working and the impact) the intervention.

The realist approach allows to study multiple systems levels (and interactions between them), for example:

- On a microlevel: interactions between social policy beneficiaries (ie, citizens, also called clients in this regard) and professionals/practitioners.
- On the mesolevel: organisational issues, such as interorganisational collaboration or accountability;
- At the macrolevel: issues of policy design.

### Actor perspective

The aforementioned underlying assumption of the realist approach states that human reactions to interventions (ie, mechanisms) differ according to circumstances (ie, context) leading to certain outcomes. Different actors on different system levels all have diverse reactions to interventions; social policy intervention not only influence citizens, but also professionals and policy-makers. In other words, by employing a realist approach, we provide transferable insights into how different actors respond in a social policy intervention, including what circumstances triggers that reaction (that can, eg, be national law for policy-makers, or overlapping programmes for professionals, or previous experiences for citizens).

### Stakeholder involvement

Involvement of stakeholders, including the population under study (ie, people receiving social assistance benefits and people facing overindebtedness) in the research process, is inherent to the realist approach.^16^

In the preparation of this study protocol, stakeholders have identified a potentially relevant intervention and provided input to refine a raw version of an initial programme theory (see online supplemental text box 1). In the project itself, stakeholders will be involved in the practical validation of the literature, identifying promising social policy interventions and in developing the initial programme theory, much like in the preparation of the proposal, but more elaborated.

### Design and methodology

#### Realist review

To develop the theoretical insights on what in social policy interventions can generate health outcomes (ie, improve gains, and prevent losses in terms of lifestyle and mental health), for whom and under what circumstances, first, a realist review is conducted. Informed by these theory-based insights from the realist review, existing relevant social policy interventions will be selected with the municipalities. Initial programme theories will be developed for the selected interventions, based on the theory-based insights from the review, combined with stakeholder insights (ie,‘dual theorising’).^19^

#### Realist evaluation

After the realist review, a realist evaluation of the selected social policy interventions will be performed. This means that the initial programme theories, consisting of multiple context-mechanism-outcome configurations, will be tested and refined or renewed if necessary. In other words, the theoretical insights from the realist review will be empirically tested and enriched.

Realist evaluation is method-neutral,^16^ which entails that methods are chosen on their suitability to empirically test the theory-based context-mechanism-outcome configurations, in order to answer the archetypical realist research question: what works, for whom and under what circumstances. Because of the complexity (ie, multiple system levels), a single method may answer only part of the research question. Therefore, we develop tailored multimethod strategies, combining multiple qualitative and/or quantitative methods, according to their contribution to answering the research question.

As suitability to test the theory-based insights depends on their actual content, and the selection of interventions in the realist synthesis, preceding the realist evaluation phase, we cannot fully define methods a priori. Potentially useful methods are illustrated in online supplemental text box 2.

#### Data analysis

Realist review and evaluation analyse literature and data retroductively. Rooted in the belief that comprehending causation requires more than just relying on observable evidence alone, retroduction combines deductive and inductive logic in a back-and-forth-movement, to identify the potential causal mechanisms that contribute to observed patterns, or variations in those patterns.^20^ When a programme theory is developed in advance, the research process begins with deductive reasoning, which involves seeking evidence to test the theory. Cases are examined, ideally reaching a point of saturation, to ensure that the observed patterns, as well as intended and unintended outcomes, align with the proposed theory. If there are inconsistent cases, it may be necessary to refine the theory. This refinement occurs through the generation of new theory based on observations or inductive reasoning. The newly formulated theory is then tested in additional cases using deductive reasoning once again.

Realist review is planned from September 2022 to December 2023. Preparations for data collection for realist evaluation (ie, stakeholders involved in decision processes) start in September 2023, actual start of data collection (ie, inclusion of participants) is in September 2024. Results of the review can be expected in 2024. Results of the evaluations can be expected in 2025, 2026 and 2027.

### Institutional approach

To develop the aforementioned governance strategies for systems change, we argue that complementary to the realist approach, an institutional approach is needed to understand and change the determining role of institutional actors in the uptake and upscaling of the realist insights.

The institutional approach is an organisational theoretical approach to understanding the interplay between institutions and institutional agents. It views actors as embedded agents, able to develop and transform existing complex systems. Organisational institutionalism focuses on how individuals themselves—as well as through (professional) groups and organisations—are intentional agents of institutional creation, maintenance and transformation.^21^

### Design and methodology

The anticipatory governance strategies will be developed in two parts. First, there will be continuous productive interactive meetings with institutional actors throughout the project duration of 4 years. We draw on theory of collaborative innovation^22^ and systemic action research^23^ to guide the institutional actors through the collaborative process. The meetings focus on building a collaboration process in which institutional actors are invited to analyse the current dynamics of the social policy system and identify obstructions and opportunities for a social system for health. In addition, the institutional actors explore during the meetings how they, each using their own position and knowledge, can work together with others towards desired changes in the social policy system.

**Figure 1:**
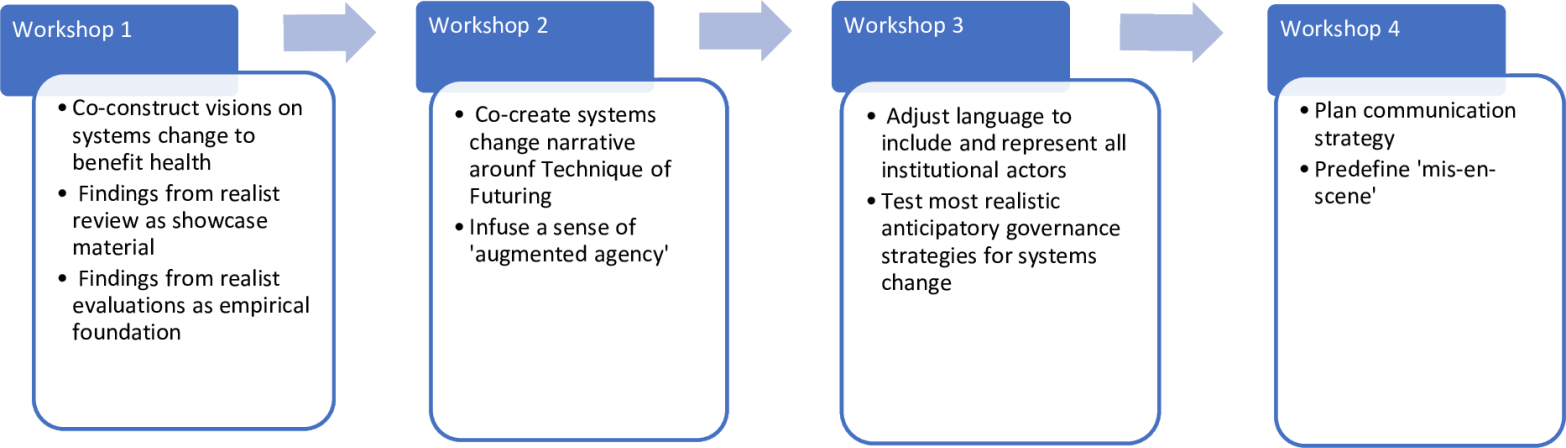
Overview of the codesigned workshops, to develop anticipatory governance strategies from an institutional approach.

Second, a series of workshops will be codesigned using techniques of futuring (ToFs).^24^ Such techniques have been proven successful in realising system-level changes in other domains, for instance, in energy transition.^25^ ToFs can be defined as ‘practices bringing together actors around one or more imagined futures and through which actors come to share particular orientations for action’(Hajer & Pelzer^25^,p225). More specifically, we achieve this by using various established tools, such as ‘whole system in the room’, stakeholder consultation and immersive design (see figure 1 for an overview of the series of workshops).

#### Stakeholder engagement

Stakeholders involved in the action research are national partners such as professional associations, national government bodies and interest associations. From the start of the research, they are invited to take an active position in contributing to system change, through their engagement in productive interactive meetings and reflecting on their own role within the system.

### Data analysis

Qualitative data, such as interviews, field notes and recordings, will be analysed according to thematic content analyses. The analysis focuses on existing and desired institutional dynamics, structures and logics, mainly expressed through narratives. To ensure quality of analysis, the following measures will be taken into account^26^:

- Interpretations will be verified with the institutional partners participating in the action research (member check).
- Multiple data sources will be used and combined in the analyses (data triangulation).
- Data coding will be discussed within the multidisciplinary research team (investigator triangulation).
  Researchers will reflect on their role and influence in the research process (reflexivity).
- Decisions and developments, and the underlying reasons, will be documented (audit trail).

Data collection and (continuous) analysis is planned from March 2023 to March 2027. Results can be expected in the course of 2026 and 2027.

### Outcomes

Priorities for reducing socioeconomic health inequalities identified in the WRR Policy Brief^5^ comprise physical activity and diet, smoking, problematic alcohol use (ie, lifestyle behaviours) and mental health in groups in vulnerable positions. The selected existing social policy interventions are evaluated on their impact on outcomes related to these priorities, for example, Statistics Netherlands microdata on antidepressant use for mental health (see online supplemental text box 2).

### Target population

We target two groups with deprivation in (economic) resources: (1) recipients of social assistance benefits (unemployment benefit of last resort) and (2) people with (a risk of) overindebtedness.

### Settings

As urban and rural settings might produce differences in vulnerability (ie, capital) and in governance strategies, having both substantially represented in the study ensures valuable insights for both science and society. In this study, two large municipalities in the strong urbanised West of the Netherlands collaborate. In the more ruralised Mid-East of the Netherlands, five municipalities of different sizes (ranging from small to mid-size) collaborate.

### Patient and public involvement

Citizens participate in the study in two ways. First, a participation board of citizens in vulnerable positions participated in the development of the study protocol. They provided research questions and ideas, and input to decide on the selection (criteria) of potential effective interventions. Moreover, they commented on the raw initial programme theory of an intervention in a (realist) focus group interview (please see online supplemental text Box 1 for an illustration). Lastly, the participation board will take part in one of the meetings with institutional actors, which focused on the citizens’ experiences of the social policy system and its effects on their health.

Second, people with mild cognitive limitations are over-represented among people in vulnerable positions, and so they are an important subtarget group. Therefore, a representative of a national interest group that gives voice to people with cognitive limitations, with lived experiences with overindebtedness and receiving social policy interventions will contribute to the research, for example, participating in the development of (communication) materials for citizens.

### Ethics and dissemination

#### Ethics

The CCMO, the Dutch Central Commission for Human Research, has assessed this study as not liable to the Medical Research Involving Subjects Act (WMO). This means that it is not required to seek ethical approval by a Medical Ethical Committee. However, advice on ethical aspects related to data collection as discussed below, will be provided by the Social Sciences ethical committee of Wageningen University. Informed consent to participate in the study will be obtained from participants for the use of all forms of personally identifiable data.

It is important that research participants are not harmed or emotionally burdened. However, data collection directly from citizens has a large added value. Their lived experiences provide relevant and accurate information on contexts, mechanisms and outcomes in the realist evaluation of selected existing social policy interventions. Data may be collected about experiences with deprivation in economic resources, social policies and (mental) health. These can be considered sensitive and stigmatised topics, possibly emotionally burdening them and causing psychological distress.

Where possible, less burdensome forms of data collection will be performed, such as using register data and document analyses. However, in case lived experiences provide indispensable insights, risk of emotional burden for participants will be diminished as much as possible, by properly preparing the methods, for example, with mock interviews.^27^ This will help to get familiar with doing interviews about a certain topic but also prepare for worst-case scenarios. This way, the researcher can learn to deal with, for example, psychological distress that can arise as a result of the research questions. The questions will also be prepared to be appropriately sensitive and avoid stigmatising language.^27^

#### Dissemination

The aim of dissemination within RASP is to reach and promote knowledge uptake among institutional actors at multiple system levels, beyond those involved in the study. The national partners involved in the action research have a large constituency among these actors and effective channels for knowledge dissemination and utilisation. Dissemination and utilisation activities will, therefore, align with these channels. At the beginning of the project, a communication plan will be formulated that maps out activities of actors involved in the study, that may offer opportunities for knowledge dissemination and utilisation. The intended target population will have a key role in determining in which form results will be presented (ie, cocreation).

We identify six types of relevant target groups for knowledge dissemination. These six target groups are listed below, together with tools, materials and activities, considered relevant for them:

#### Citizens with (risk of) problematic debts and/or receiving social assistance benefits

Communication materials will be developed in cocreation with citizens in vulnerable positions, including a representative from the interest group for people with mild cognitive limitations. An example is an icon folder or videoclip.

#### Students

Educational materials for students of social work, and social and legal services will be developed in cocreation with students, lecturers and current professionals, to ensure fitting of the material in the education programme and the daily practice, such as an e-learning module or a guest lecture.

#### Professionals working in public services related to social policy

The publications in professional journals that are planned in this project, target mainly the executing professionals.

#### Policy-makers and managers in municipal public services-related social policy and/or public health

This target group will be reached through different channels and in different forms; factsheets and infographics will be provided to share the direct insights related to the research, contributions will be made to existing communication channels, such as journals, podcasts and blogs, recurrent symposium. In addition, an end-symposium will be organised.

#### Policy-makers in the national government, working in public health or social policy domain

This target group will be reached throughout the project as representatives of this group actively take part in the action research component, as well as at the end of the project through an (online) symposium.

#### Scientific community

This community will be reached through scientific publications and presentations at relevant conferences, such as the international debt research group that meets annually at the Law and Society Association Conference.

In addition to the specified target populations, the general audience will be informed about milestones in the project by issuing press releases, and/or LinkedIn messages, and/or corporate communication channels.

## Discussion

A first strength can be found in the cross-domain systems approach to health inequalities. Many of the various factors influencing health, such as social, economic, environmental and commercial aspects, which are vital for safeguarding and fostering population health, are in fact not part of the traditional health domain.^28^ Therefore, the WHO^28^ argues to expand the boundaries of what are considered systems that contribute to population health beyond the traditional health systems, into ‘systems for health’. In this perspective, social policy systems are considered to be (potential) systems for health.

A strength of this study protocol is also that it enables both an in-depth understanding of how social policy contributes to health outcomes in citizens in vulnerable positions, and a strategical preparation for uptake and upscaling of these insights. To better understand the mechanisms through which social policy generate health outcomes, and under which contextual circumstances these mechanisms can fire, the project conducts a realist evaluation of existing policy interventions embedded in local social policy systems. To strategically prepare for uptake and upscaling of social policy interventions, the project takes an institutional approach to develop such strategies. The combination of these two complementing approaches is both innovative as well as suitable for the required systems change.

Another strength can be found in the settings of this study protocol. Both the forms and causes of vulnerability in populations and social policy practices (including means and possibilities) vary between municipalities, and across rural and urban settings. Both settings are represented in this study by multiple municipalities, varying in size.

A last strength can be found in the transdisciplinarity of this study protocol. To get a full understanding of the workings of social policies, a transdisciplinary team that works in close cooperation is required. The realist approach is particularly appropriate as a catalyst for achieving synergy between disciplines, as it brings together theory in a transdisciplinary way, and methods from different disciplines complement each other. (Behavioural) health sciences is a multidisciplinary field of study, related to health and healthcare. Especially relevant to the project is expertise from this field of study on (determinants and psychological pathways of) lifestyle behaviours but also expertise to evaluating complex interventions. Sociology contributes expertise to the study of inequality in access to diverse forms of resources as well as the contextual nature of social phenomena. Empirical microeconomics adds expertise to microeconomic analyses of behaviour of households, with regard to household finance, employment and health outcomes. Governance and organisational science adds expertise to the development of implementation strategies, drawn from institutional approaches. Furthermore, the realist approach can be considered an integrative approach, as stakeholders are involved throughout the research process, including dual theorising (ie, integrating scientific theories with stakeholder theories^29^) and validation of results. In other words, in this project, scientific knowledge is combined with professional knowledge and experiential knowledge.

Although the cross-domain approach in this study can be considered promising, health inequalities are a genuine so-called wicked problem. Over the past decennia, despite multiple efforts, the gap in life expectancy between the wealthiest, and the least wealthiest group in the Netherlands, have only grown.^5^ It can be questioned whether social policy intervention can result in sufficient health gains to genuinely reduce health inequality. Possibly, the potential benefit of social policies lies in protecting the current health status of citizens in vulnerable positions (ie, no further exacerbation of their health status), rather than actually promoting it (ie, no actual increase in healthy lifestyle behaviour and improving mental health). However, given the current trend of the ever-growing gap, no further exacerbation might then be still considered a relevant step. It might be, that in order to reach health inequality through actual health promotion, not only the social policy domain should be involved, but, for example, also other policy domains such as the built environment (eg, the food environment, the physical activity environment).

Another limitation of the cross-domain approach might be that some might consider evaluating health impact of policies that have other objectives (such as disposing of sufficient resources) as a form of ‘health imperialism’. However, in this study, the primary objectives of social policies (ie, redistribution of resources) are considered a crucial part of the pathway to health impact. Health is not considered more important than disposing of sufficient resources, but the bidirectional relation of health and disposing of resources is considered crucial for health inequalities. As such, this study protocol aligns with the health for all policies—development.^29^

Another potential limitation that can be found in reaching the objective of this study is the challenge that social policy (ie, how should burdens and resources be distributed in society, and who is deserving of what?) is subject to political ideology. This particularly might play a role in the action research, for example, in the willingness to change. Additionally, the political climate itself might change, which, through institutional design, might lead to a challenge for support among stakeholders.

## Supplementary Material

Reviewer comments

Author's manuscript
