# Supplementary material for: Realist Approach to Social Policies (RASP) study to reduce socioeconomic health inequalities through systems change: protocol for a research project combining mixed-methods realist research with institutional action research
Source: BMJ Open. 2024 Jun 13;14(6):e088571. doi: 10.1136/bmjopen-2024-088571 (PMC11177664; doi:10.1136/bmjopen-2024-088571)
Supplement: Supplementary data [file bmjopen-2024-088571supp001.pdf]

Supplemental File 1 STATE OF THE ART: SOCIAL POLICY AND HEALTH

In this section, we summarize the state of the art of research on the effects of social policy on health. In general, the underlying mechanisms and transferability of findings to a Dutch-type welfare context often remains unclear. Looking at the Netherlands, there are even substantial differences between regions (urban and rural) and municipalities. In line with the two main target groups for this project, namely people who receive social assistance and people facing problematic debt, we summarize the state of the art of the health impact of two types of social policy that they are subject to, namely social benefits policy, and debt policy. We tailor our summary towards dimensions of social policies that can be modified in social policy interventions and that point towards opportunities for improved realization of health potential. Strikingly, there is much more known about social assistance policy than debt policy. Research on the Netherlands is scarce in general and there is little to no research that takes an explicit complex systems approach.

Impact of Social Assistance Benefits on Health Outcomes

The literature distinguishes three relevant dimensions of social benefit policies that might affect health outcomes: 1) generosity and eligibility, 2) active labour market policies, and 3) requirements, monitoring and sanctions. Considering 1), in the Netherlands, social assistance is a non-contributory, monthly transfer scheme of last resort for unemployed people who do not or no longer qualify for other benefit schemes, including unemployment benefits. Eligibility for this scheme is based on a work- and means-test, while the benefit level depends on the household composition. In view of 2) labour and reintegration requirements in the Netherlands including active search for a job, willingness to accept all types of paid employment offered, participation in active labour market programs, following education and skill-development programs, signing up with a temporary employment agency (if requested), and performing volunteer work. Lastly 3), as is common to such schemes (1), social assistance claimants have to comply with regulations and obligations, such as

administrative requirements (e.g., providing information, attending appointments, and responding to letters and emails) to maintain (full) entitlement to the benefit.

1. Effects of generosity and eligibility of social benefit systems

Findings of a recent literature review show that widened eligibility and increased generosity of social benefit systems are associated with improved mental health and reduced mental health inequalities (2). Similarly, reduced eligibility and generosity were associated with deteriorated mental health and increased mental health inequalities.

These latter findings were in line with findings of a literature review examining the health effect of social assistance programs (3). This review shows that stricter eligibility and generosity result in worse health outcomes. Simpson et al. (2) distinguish mechanisms at two levels that might underly these effects: 1) at the aggregate level (e.g., lower generosity and stricter eligibility criteria coincide with stigmatizing attitudes toward benefit claimants and increased income inequality which may subsequently impair mental health) and 2) at the individual level (e.g., increased generosity and eligibility may hamper employment prospects of claimants which subsequently impair mental health). The direction of the effects of expansionary and contractionary policies on mental health is not straightforward as these policies might trigger mechanisms both improving and harming mental health. The underlying mechanisms are complex and might depend on moderating factors.

However, widened eligibility may not improve health in all contexts. In the Netherlands, a randomized experiment among social assistance claimants in The Netherlands shows that imposing a 1-month job search period upon applicants lowered welfare take-up and increased employment rates, while this stricter eligibility regulation did not affect (mental) health (4).

2. Effects of active labor market policies (ALMPs)

A second aspect of social benefits policy that is relevant for health outcomes are Active Labor Market Policies (ALMPs). ALMPs aim to increase re-employment rates and include job search assistance, job or vocational training, and subsidized public and private employment. In general, literature reviews and meta-studies have found that ALMPs have small effects on re-employment in the short run, but larger effects in the medium and long run (e.g., (5,6). Job search assistance programs focusing on 'work first' have similar effects in the short and long run, while job training and private sector employment programs increase employment only in the medium and longer run (probably due to lock in effects).

A literature review about the effects of ALMPs on self-reported health outcomes showed that job search assistance including a psychological component (e.g., enhancing self-efficacy) have positive effects on mental health (7). In general, high-risk groups benefitted most. Effects of vocational training programs and subsidized employment were mostly positive but reflected more diversity. In a recent study using administrative data, Caliendo et al. (8) found that participation in a vocational training program reduced drug prescriptions for cardiovascular and mental health problems by about 6-8 percent, while sickness absence reduced by about 20 percent. This effect was larger for vulnerable groups (e.g., lower educated claimants). Effects were likely to be direct (via adoption of a daily routine) rather than indirect (via improved employment prospects) as the reduction in drugs prescription started when claimants were still enrolled in the training program.

Findings in the Netherlands are generally consistent with international evidence. As found in the Dutch social assistance context, entering paid employment has a positive impact on mental and physical health (9). As a consequence, active labor market policies might act as important health promotion policies among unemployed individuals. In the Dutch social assistance context, welfare departments usually provide employment services (including job search assistance and regular encounters with caseworkers) following a target-group based approach. Schuring et al. (10) investigated the effects of different types of employment service approaches for Dutch social

assistance claimant using a quasi-experimental approach. They found that Matching (approach focusing on facilitating fast re-employment) reduced drug prescriptions for mental health problems by six percentage points compared to Pre-matching (focus on reducing labor market barriers). These approaches were not significantly different in their impact on other health outcomes (cardiovascular diseases, diabetes, and respiratory illness). Matching also resulted in higher re-employment rates than Pre-matching which might explain the positive finding on mental health. These findings suggest that ALMPs could be important to realize a structural breakthrough in improving mental health among vulnerable individuals.

3. Effects of requirements, monitoring, and sanction policy

A third aspect of social benefits policy that can affect health outcomes is the enforcement regime. Economic studies have shown that stricter labor and reintegration requirements, increased monitoring, and more stringent sanction policy tend to decrease unemployment duration and increase job entry rates of (sanctioned) claimants (11–14), although effects often disappear in the long run and these policies might reduce post-unemployment job quality (15–17).

Only a few studies investigated the impact of (elements off) the enforcement regime on health outcomes. Some qualitative and correlational studies conducted in the UK suggest that stricter labour and reintegration requirements, monitoring, and sanction policy are predominantly experienced as stressful and punitive and may negatively affect the mental health and well-being of claimants (18–20). Among claimants of a Danish unemployment benefit program, Baekgaard et al. (21) found that reducing compliance demands to labour and reintegration requirements lowered reported stress levels and increased autonomy. They did not find an effect on stigma. Caliendo et al. (8) investigated the effects of imposed benefit sanctions due to non-compliance to labour and reintegration requirements. They found an increase in drug prescriptions for mental health problems in the month before sanction was imposed (probably due to receiving a warning) and an increase in sickness absence in the months after the sanction was imposed. They did not find a long-run effect

on drug prescriptions related to cardiovascular diseases and mental health problems. A potential explanation is that negative health effects of being sanctioned due to financial stress might be compensated by a positive health effect via increased re-employment rates. Understanding the mechanisms in the Dutch context would help to design effective policies with positive health effects both in the short and the long run.

Impact of Debt Policy on Health Outcomes

In comparison with studies on social assistance benefits policies, studies investigating the effects of debt policy or interventions on health are scarce. We can distinguish three dimensions of debt policy for potential health outcomes: 1) debt counselling, 2) altering the composition of creditors, and 3) debt restructuring and reduction. In the Netherlands, those dimensions are highly interrelated in debt policy. People who are unable to repay their debts on their own can apply for voluntary or statutory debt restructuring based on the law *Wet schuldsanering natuurlijke personen* (Wsnp) (translated into Natural Persons Debt Rehabilitation Act). Municipalities appoint debt counsellors who develop a voluntary payment arrangement based on a calculation of how much the debtor can pay to the creditors each month over a period of three years. If the creditors accept the voluntary payment plan, and the debtor adheres to the plan without entering new debts, the remaining debt is acquitted after three years. If the creditors fail to agree to the payment plan, the citizen can apply for statutory debt restructuring. If the application is granted, the judge appoints an administrator who oversees the process and creditors are obliged to agree with the payment plan. Similar to a voluntary plan, after three years of payments, remaining debts are absolved. Despite this apparent protection offered to people with problematic debts under the law, only a fraction (ca. 14%) of eligible individuals actually seek assistance (22). To make matters worse, the stabilization process that precedes the voluntary repayment plan often takes months and has a high dropout rate. In addition, municipal approaches are often not well tailored to the complex realities of the lives of

people with problematic debts, and creditors are not always cooperative. Municipalities differ in the conditions and requirements they impose. For example 20% of the municipalities ask for documentation of the financial situation prior to enrolment, which can be a significant burden for people in problematic debt. It is safe to state that debt policy now fails to reach the large majority of this vulnerable target group, though there are mayor differences between municipalities.

1. Debt counselling

Of the three dimensions, most evidence is available on the effect of debt counselling on health. A cross-sectional study found that debt counselling significantly reduced financial stressor events among its clients, which in turn was associated with a more positive perception of one's financial situation and perceived health. There were no significant direct effects of debt counselling on perceived health, possible due to the follow-up period of 18 months being too short (23). Similarly, a literature review on the effects of financial counselling shows that there is little evidence for the effect of financial counselling on health. This may be explained by the characteristics of interventions. Most of the interventions that were evaluated put great emphasis on knowledge, while applying behavioural insights might increase the effectiveness of counselling (24). One example of an intervention used in debt counselling that incorporates several behavioural insights is Mobility Mentoring, which was developed by the US-based organization EMPath. Mobility Mentoring has shown promising initial results in the US, but has yet to be proven effective (25). There is scarce evidence in the Netherlands on the effect of debt counselling on client health. A process evaluation of Mobility Mentoring at a Dutch municipality showed that after six months of assistance, clients reported that their health problems were less hindering for their participation in caretaker, educational or work-related activities. One study compared three types of debt counselling In the Netherlands (26). Intensive support across multiple life domains for clients with complex problems, but also target financial counselling combined with administrative and legal support for relatively self-sufficient clients were both suggested to be successful. Success in

addressing financial problems coincided with less stress and more belief in a positive outcome in both groups. A counselling type with more limited support accessible for all client types generated less success and less positive health outcomes. Clients experience myriad of emotions, for which there is little attention in the counselling sessions(26). The authors believe that more attention for the client's experience may reduce the attrition rate of debt restructuring trajectories.

2. Altering the composition of creditors

There is evidence that a smaller number of creditors might have positive health effects. A quasi-experimental study compared households in Singapore that received a certain amount of money to pay off their debts (27). All households received the same amount, which translated into different numbers of creditors that were paid off. Each additional creditor that was paid off was associated with an improved cognitive functioning by a quarter of standard deviation, and 11% less anxiety and 10% less present bias. A qualitative study among UK citizens showed that borrowing from microcredit providers was perceived as having positive effects on health (28). Micro credit reduced stress in the face of unexpected financial costs; it enabled them to maintain social relationships and increased the perceived control over their lives.

3. Debt restructuring and reduction

In a qualitative study on re-employment among participants of debt restructuring programs, participants reported that the start of the voluntary payment plan led to less mental and physical health complaints, such as stress, sleeping problems, and feelings of depression (29).

GAPS IN KNOWLEDGE

While there are some promising leads for reducing health inequalities through social policy, there is still much to be clarified. As discussed, studies investigating the health effects of debt policies are

scarce. The literature reviews on social assistance benefit policies report or show a lack of studies conducted in a Dutch-type welfare context.

In addition, there is a lack of studies unravelling the mechanisms underlying the effects of social policies on health outcomes. As a consequence of these gaps, we should be cautious in generalizing the results to the Dutch social assistance benefits context. The present research project aims to fill these gaps for both policy domains. In general, health impacts are usually an unintended by-product of policies that are themselves complex and such effects are embedded and possibly counteracted in a complex system (30). In addition, studies usually fail to account for contextual differences (e.g., rural and urban settings) and to address both ex-ante and ex-post effects of social policies. An approach is necessary that combines evidence on impacts, but that also accounts for the way in which contextual variables interact with policy.

REFERENCES

1. Immervoll,H. K C. How demanding are activation requirements for jobseekers [Internet]. 2018 Jul [cited 2023 Oct 2]. (OECD Social, Employment and Migration Working Papers; vol. 215). Report No.: 215. Available from: https://www.oecd-ilibrary.org/employment/how-demanding-are-activation-requirements-for-jobseekers_2bdfecca-en
2. Simpson J, Albani V, Bell Z, Bambra C, Brown H. Effects of social security policy reforms on mental health and inequalities: A systematic review of observational studies in high-income countries. Soc Sci Med. 2021 Mar;272:113717.
3. Shahidi FV, Ramraj C, Sod-Erdene O, Hildebrand V, Siddiqi A. The impact of social assistance programs on population health: a systematic review of research in high-income countries. BMC Public Health. 2019 Dec;19(1):2.
4. Bolhaar J, Ketel N, Van Der Klaauw B. Job Search Periods for Welfare Applicants: Evidence from a Randomized Experiment. Am Econ J Appl Econ. 2019 Jan 1;11(1):92–125.
5. Card D, Kluve J, Weber A. What Works? A Meta Analysis of Recent Active Labor Market Program Evaluations. J Eur Econ Assoc. 2018 Jun 1;16(3):894–931.
6. Vooren M, Haelermans C, Groot W, Maassen Van Den Brink H. THE EFFECTIVENESS OF ACTIVE LABOR MARKET POLICIES: A META-ANALYSIS. J Econ Surv. 2019 Feb;33(1):125–49.
7. Puig-Barrachina V, Giró P, Artazcoz L, Bartoll X, Cortés-Franch I, Fernández A, et al. The impact of Active Labour Market Policies on health outcomes: a Scoping review. Eur J Public Health [Internet]. 2019 Mar 25 [cited 2023 Oct 2]; Available from: https://academic.oup.com/eurpub/advance-article/doi/10.1093/eurpub/ckz026/5419277
8. Caliendo M, Mahlstedt R, Van Den Berg GJ, Vikström J. Side Effects of Labor Market Policies. SSRN Electron J [Internet]. 2020 [cited 2023 Oct 2]; Available from: https://www.ssrn.com/abstract=3730444
9. Schuring M, Robroek SJ, Burdorf A. The benefits of paid employment among persons with common mental health problems: evidence for the selection and causation mechanism.

Scand J Work Environ Health [Internet]. 2017 Oct 2 [cited 2023 Oct 2]; Available from: http://www.sjweh.fi/show_abstract.php?abstract_id=3675
10. Schuring M, Knoef M, Burdorf A. The effect of employment services on employment and health among unemployed persons: an IV approach. Eur J Public Health. 2022 Oct 21;32(Supplement_3):ckac131.263.
11. Abbring JH, Berg GJ, Ours JC. The Effect of Unemployment Insurance Sanctions on the Transition Rate from Unemployment to Employment. Econ J. 2005 Jul;115(505):602–30.
12. Arni P, Schiprowski A. Job search requirements, effort provision and labor market outcomes. J Public Econ. 2019 Jan;169:65–88.
13. Svarer M. The Effect of Sanctions on Exit from Unemployment: Evidence from Denmark: Effect of Sanctions on Exit from Unemployment. Economica. 2011 Oct;78(312):751–78.
14. Van Den Berg GJ, Van Der Klaauw B, Van Ours JC. Punitive Sanctions and the Transition Rate from Welfare to Work. J Labor Econ. 2004 Jan;22(1):211–41.
15. Arni P, Lalive R, Van Ours JC. HOW EFFECTIVE ARE UNEMPLOYMENT BENEFIT SANCTIONS? LOOKING BEYOND UNEMPLOYMENT EXIT: BENEFIT SANCTIONS BEYOND UNEMPLOYMENT EXIT. J Appl Econom. 2013 Nov;28(7):1153–78.
16. Lachowska M, Meral M, Woodbury SA. Effects of the unemployment insurance work test on long-term employment outcomes. Labour Econ. 2016 Aug;41:246–65.
17. Van Den Berg GJ, Vikström J. Monitoring Job Offer Decisions, Punishments, Exit to Work, and Job Quality. Scand J Econ. 2014 Apr;116(2):284–334.
18. Dwyer P, Scullion L, Jones K, McNeill J, Stewart ABR. Work, welfare, and wellbeing: The impacts of welfare conditionality on people with mental health impairments in the UK. Soc Policy Adm. 2020 Mar;54(2):311–26.
19. Wright S, Fletcher DR, Stewart ABR. Punitive benefit sanctions, welfare conditionality, and the social abuse of unemployed people in Britain: Transforming claimants into offenders? Soc Policy Adm. 2020 Mar;54(2):278–94.
20. Wright S, Patrick R. Welfare Conditionality in Lived Experience: Aggregating Qualitative Longitudinal Research. Soc Policy Soc. 2019 Oct;18(4):597–613.
21. Baekgaard M, Mikkelsen KS, Madsen JK, Christensen J. Reducing Compliance Demands in Government Benefit Programs Improves the Psychological Well-Being of Target Group Members. J Public Adm Res Theory. 2021 Sep 13;31(4):806–21.
22. Schonewille, G. & Weijers, M. Veel schuldenaren weten schuldhulp slecht te vinden. ESB. 2019;104(4776).
23. Kim, Jinhee and Garman, E. Thomas and Sorhaindo, Benoit,. Relationships Among Credit Counseling Clients' Financial Wellbeing, Financial Behaviors, Financial Stressor Events, and Health [Internet]. Journal of Financial Counseling and Planning; 2003. Available from: https://ssrn.com/abstract=2265623
24. Jungmann, N., Madern, T. Duurzame verandering van financieel gezond gedrag: Droom of werkelijkheid? [Internet]. WRR; 2016. Available from: https://www.wrr.nl/publicaties/working-papers/2016/06/30/duurzame-verbetering-van-gezond-financieel-gedrag.-droom-of-werkelijkheid#:~:text=Droom%20of%20werkelijkheid%3F%20is%20uitgevoerd,gezonder%20financieel%20gedrag%20gaan%20vertonen.
25. Washington State Department of Early Learning. Year one report - ECEAP Family Support Pilot june 2015 -june 2016 [Internet]. Olympia; 2016. Available from: https://dcyf.wa.gov/sites/default/files/pdf/eceap/ECEAP_Family_Support_Pilot_Report_Final.pdf

26. van Logtestijn, B. van der Want,E. van Jeveren, F., Dirven, J. & Broere, M. Gemeenten zetten de schouders eronder [Internet]. 2019. Available from: https://vng.nl/publicaties/gemeenten-zetten-de-schouders-eronder
27. Ong Q, Theseira W, Ng IYH. Reducing debt improves psychological functioning and changes decision-making in the poor. Proc Natl Acad Sci. 2019 Apr 9;116(15):7244–9.
28. Myrskylä M, Gagnon A, Bengtsson T. Pathways to Health and Well-being. Soc Sci Med. 2014 Oct;119:175–9.
29. van der Laan, J. & van Geuns, R. Schuldenvrij: De weg naar werk? [Internet]. 2016. Available from: https://www.hva.nl/kmr/gedeelde-content/publicaties/publicaties-algemeen/2016/schuldenvrij-de-weg-naar-werk.html
30. Shiell A, Hawe P, Gold L. Complex interventions or complex systems? Implications for health economic evaluation. BMJ. 2008 Jun 7;336(7656):1281–3.

Supplemental Text box 1 ILLUSTRATION OF A SOCIAL POLICY INTERVENTION AND ITS INITIAL

"Vroeg Eropaf" (Early Outreach) is a debt policy intervention designed to address starting problematic debts through a multi-component approach. This illustration provides an overview of the intervention, presents the initial program theory, and outlines the proposed research methodology for testing and refining the theory.

The Vroeg Eropaf intervention aims to identify and address starting problematic debts through three key components. Firstly, a network of service providers signals arrears in payment to municipalities when individuals experience financial difficulties. Secondly, social service teams from municipalities make contact with households when at least two service providers signal arrears, offering assistance if needed. Lastly, if assistance is desired, the teams provide support in managing financial problems by assigning individuals to debt service departments or other societal organizations. This intervention adheres to Dutch and European privacy regulations, and Dutch municipalities have a legal duty to engage in early signaling of debt since January 1st, 2021. Vroeg Eropaf is currently implemented in multiple municipalities, both in urban and rural settings, and has been identified as a potentially relevant intervention for this research.

The initial program theory is informed by existing literature on the impact of overindebtedness on health outcomes. Studies have shown a correlation between debt and various mental health issues, including mental disorders, depression, suicide attempts, problem drinking, drug dependence, neurotic disorders, and psychotic disorders. The underlying mechanisms linking overindebtedness to poor health involve worry, financial scarcity, stress, negative affect, social isolation, reduced perceived control, reduced sleep quality, failure to seek assistance, poor medication adherence, impaired executive functioning, and unhealthy lifestyle behaviors such as unhealthy eating, smoking, and reduced physical activity.

Furthermore, the theory proposes that the reduction of administrative burdens, which individuals may experience in their interactions with the state, plays a significant role in improving health outcomes. Administrative burdens consist of learning costs (searching for information about social programs), compliance costs (meeting administrative requirements), and psychological costs (stress, frustration, loss of autonomy, and stigma). The assistance component of Vroeg Eropaf aims to alleviate these burdens, potentially reducing barriers to accessing debt services and improving overall well-being.

However, it is important to note that the intervention may not have a positive impact on health outcomes for certain groups or under specific conditions. Challenges may arise in effectively reaching all citizens with starting problematic debts, especially if the network of service providers fails to adequately signal or if there are capacity limitations or barriers related to language, literacy, or cultural differences. Additionally, behavioral changes, such as improved financial behavior or increased uptake of debt services, may not occur for some approached individuals due to unproductive interactions with professionals or a lack of trust.

Stakeholder involvement is a crucial aspect of this research, employing 'dual theorizing' by consulting policy advisors and citizens in vulnerable positions. Stakeholders recognize the potential relevance of Vroeg Eropaf for improving health outcomes. Citizens highlighted the interaction between mental health and their response to the intervention, emphasizing that poor mental health can hinder help-seeking behavior. Other stakeholders also acknowledged the significance of administrative burden, considering it a sort of "hygiene condition" that, when present, exacerbates individuals' situations without guaranteeing improvement upon its reduction. Policy advisors expressed an awareness of the burdens placed on citizens and sought alternatives to alleviate them. In a rural municipality, the concept of 'noaberschap' (neighborship), a form of social resource rooted in historical tradition, was identified as potentially impacting the intervention's outcomes by either triggering feelings of shame and hampering health outcomes, or strengthening social resource and improving health outcomes.

PROGRAM THEORY

Supplemental Text box 2 ILLUSTRATION OF A MULTI-METHOD EVALUATION STRATEGY

A mixed-method evaluation strategy is utilized to explore various dimensions and levels within the program theory. A combination of document analyses, quasi-experimental design, realist interviews and ethnographic institutional analysis is under consideration for the evaluation of "Vroeg Eropaf" (Early Outreach, see text box 1).

Document analysis entails a meticulous examination of the intervention's implementation and its reach in the municipality under study. This analytical process serves as the foundation for understanding relevant micro, meso, and macro levels and dimensions to be investigated.

We could estimate effects of the Vroeg Eropaf-intervention on resource-related (e.g., labor status and debt situation) and health-related (mental health, physical health) outcomes. To gain insight in the heterogeneity of intervention effects, we will additionally examine whether intervention effects differ among subgroups (e.g., high-risk groups related to (mental) health problems). To identify these intervention effects, we **link administrative data of municipalities to register data of Statistics Netherlands**. Register data of Statistics Netherlands provide individual- and household-level data for all Dutch residents. The data covers several topics ranging from demographics, education, and labour market status to prescribed medication and problematic debts.

Linking municipality data to this data register of Statistics Netherlands provide a unique opportunity to investigate the outcomes of (local) social policy interventions. Recent studies have shown that linking these data sources is feasible and appropriate to identify intervention outcomes of social policy interventions (e.g., 19, 20). To estimate intervention outcomes, we can use quasi-experimental methods, such as instrumental variable approaches, difference-in-difference analyses, regression discontinuity designs, and propensity score matching. These methods fit realist evaluation, as it allows to test the transfactuality of Context-Mechanism-Outcome configurations (60). An appropriate method will be chosen depending on the context characteristics and the availability of data.

Furthermore, on a microlevel, we can investigate the workings of the aforementioned potential mechanisms such as administrative burden, and the contexts that trigger (or prevents them) the mechanisms, such noaberschap in a rural municipality, through **(realist) interviews** with citizens.

Ethnographic institutional analysis of the collaboration between institutional actors allows to explore how micro patterns of professional behavior play a role in the intervention on a mesolevel, and to explore systems-design issues on a macro-level.

Ultimately, this comprehensive combination of methods is aimed at refining, refuting, or revising the context-mechanism-outcome configurations within the program theory, underpinning the workings of the social policy intervention.
