# Supplementary material for: Realist Approach to Social Policies (RASP) study to reduce socioeconomic health inequalities through systems change: protocol for a research project combining mixed-methods realist research with institutional action research
Source: BMJ Open. 2024 Jun 13;14(6):e088571. doi: 10.1136/bmjopen-2024-088571 (PMC11177664; doi:10.1136/bmjopen-2024-088571)
Supplement: Author's manuscript [file bmjopen-2024-088571.draft_revisions.pdf]

BMJ Open

BMJ Open is committed to open peer review. As part of this commitment we make the peer review history of every article we publish publicly available.

When an article is published we post the peer reviewers' comments and the authors' responses online. We also post the versions of the paper that were used during peer review. These are the versions that the peer review comments apply to.

The versions of the paper that follow are the versions that were submitted during the peer review process. They are not the versions of record or the final published versions. They should not be cited or distributed as the published version of this manuscript.

BMJ Open is an open access journal and the full, final, typeset and author-corrected version of record of the manuscript is available on our site with no access controls, subscription charges or pay-per-view fees (http://bmjopen.bmj.com).

If you have any questions on BMJ Open's open peer review process please email info.bmjopen@bmj.com

BMJ Open

The Realist Approach to Social Policies (RASP) study: A study protocol for mixed-methods realist research combined with institutional action research, to reduce socioeconomic health inequalities through systems change

Journal:	*BMJ Open*
Manuscript ID	bmjopen-2024-088571
Article Type:	Protocol
Date Submitted by the Author:	09-May-2024

Complete List of Authors:	Van Berkel, Jantien; Utrecht University, Interdisciplinary Social Science: Public Health; Wageningen Universiteit en Research, Consumption and Healthy Lifestyles<br>de Bruijn, Ernst-Jan; Leiden University, Institute of Tax Law and Economics | Department of Economics<br>Waardenburg, Maikel; Utrecht University, Law, Economics and Governance; Utrecht University School of Governance<br>la Grouw, Yvonne; Utrecht University, Law, Economics and Governance; Utrecht University School of Governance<br>van Bennekom, Eline; Wageningen Universiteit en Research, Consumption and Healthy Lifestyles; Utrecht University, Interdisciplinary Social Science: Public Health<br>van der Horst, Hilje; Wageningen Universiteit en Research, Consumption and Healthy Lifestyles<br>Tonnon, Susanne; Hogeschool Utrecht, Debt and Collection<br>Haggenburg- Mohammed, Milou; Gemeente Amsterdam, Onderzoek en Statistiek<br>Haveman-Nies, Annemien; Wageningen Universiteit en Research, Consumption and Healthy Lifestyles; GGD Noord en Oost Gelderland, AGORA Academische werkplaats<br>Madern, Tamara; Hogeschool Utrecht, Debt and Collection<br>Knoef, Marike; Tilburg University, Tilburg School of Economics and Management; Leiden University, Institute of Tax Law and Economics | Department of Economics<br>de Vet, Emely; Tilburg University, Tilburg School of Humanities and Digital Sciences; Liberal Arts and Sciences; Wageningen Universiteit en Research, Consumption and Healthy Lifestyles

Keywords:	Health Equity, Community-Based Participatory Research, MENTAL HEALTH, Overweight, Smoking Reduction

SCHOLARONE™ Manuscripts

Title page

The Realist Approach to Social Policies (RASP) study: A study protocol for mixed-methods realist research combined with institutional action research, to reduce socioeconomic health inequalities through systems change

Authors, email addresses and affiliated institutions

Jantien van Berkel, j.vanberkel@uu.nl [1,2]

Ernst-Jan de Bruijn, e.de.bruijn@law.leidenuniv.nl [3]

Maikel Waardenburg, M.Waardenburg@uu.nl [4]

Yvonne la Grouw, y.m.lagrouw@uu.nl [4]

Eline van Bennekom, eline.vanbennekom@wur.nl [2,1]

Hilje van der Horst, Hilje.vanderhorst@wur.nl [2]

Susanne Tonnon, susanne.tonnon@hu.nl [5]

Milou Haggenburg- Mohammed, m.haggenburg@amsterdam.nl [6]

Annemien Haveman, annemien.haveman@wur.nl [2,7]

Tamara Madern, tamara.madern@hu.nl [5]

Marike Knoef, M.G.Knoef@tilburguniversity.edu [8]

Emely de Vet, E.W.M.L.deVet@tilburguniversity.edu [9]

For peer review only

For peer review only - http://bmjopen.bmj.com/site/about/guidelines.xhtml

1 Utrecht University | Department of Interdisciplinary Social Science | Padualaan 14, 3584 CH Utrecht |The Netherlands

2 Wageningen University and Research | Chairgroup Consumption and Healthy Lifestyles | Hollandseweg 1, 6706 KN Wageningen | The Netherlands

3 Leiden University | Department of Economics| Steenschuur 25, 2311 ES Leiden | The Netherlands

4 Utrecht University| School of Governance | Bijlhouwerstraat 6-8, 3511 ZC Utrecht | The Netherlands

5 University of Applied Sciences Utrecht | Research Group Debt and Collection| Padualaan 101, 3584 CH Utrecht | The Netherlands

6 Gemeente Amsterdam | Department of Research and Statistics | Oudezijds Voorburgwal 300, 1012 GL Amsterdam| The Netherlands

7 GGD-Noord- en Oost Gelderland| Rijksstraatweg 65, 7231 AC Warnsveld| The Netherlands

8 Tilburg University | Tilburg School of Economics and Management | P.O. box 90153, 5000 LE Tilburg| The Netherlands

9 Tilburg University| Tilburg School of Humanities and Digital Sciences | PO Box 90153, 5000 LE Tilburg | The Netherlands

Corresponding author: Jantien van Berkel

Postal address: P.O. box 80140, 3508 TC Utrecht

E-mail adress: j.vanberkel@uu.nl

Telephone: 06-48115026

Key words: health inequality; social policy; realist evaluation; participatory action research

Word count: 7690 words

For peer review only

Abstract

Introduction

Health inequalities are rooted in inequality in vital resources for health, including financial resources, a supportive informal network, a stable living situation, work or daytime activities, or education and literacy. About 25% of Dutch citizens experience deprivation in such resources. Social policy consists of crucial instruments for improving resources in those groups, but can also have adverse effects and lead to additional burden.

This project aims to contribute to the reduction of health inequalities through 1) better understanding how social policy interventions can contribute to reducing health inequality through the redistribution of burdens and resources, and 2) developing anticipatory governance strategies to implement those insights, contributing to a change in social policy systems.

Methods

Two systems approaches are combined for establishing a systems change. Firstly, a realist approach enables insights into what in social policy interventions may impact health outcomes, for whom, and under what circumstances. Secondly, an institutional approach enables scaling up these insights, by acknowledging the crucial role of institutional actors for accomplishing a systems change.

Together with stakeholders, we perform a realist review of the literature and identify existing promising social policy interventions. Next, we execute a realist evaluation of selected social policy interventions in seven municipalities, ranging from small, mid-size to large, and in both urban and rural settings. Simultaneously, through action research with (national) institutional actors, we facilitate development of anticipatory governance strategies.

Ethics and Dissemination

The main ethical challenges of this project relate to the vulnerable positions of the populations under study. Extra attention is paid to prevent additional emotional burdening in the research

For peer review only

process. We co-develop knowledge products and activities, with the targeted professionals, students, citizens and governments, to stimulate uptake of the knowledge produced in the project.

For peer review only

For peer review only - http://bmjopen.bmj.com/site/about/guidelines.xhtml

Article Summary

Strengths and limitations of this study

- The cross-domain systems approach expands understanding beyond traditional health systems by integrating social policy as potential systems for health.
- Realist evaluation provides insight into how social policy contributes to health outcomes and prepares for strategic uptake and upscaling.
- Diverse settings are represented, including both urban and rural municipalities, capturing variations in vulnerability and social policy practices.
- The "health imperialism" critique suggests that evaluating policies primarily focused on resource distribution may raise ethical concerns about the prioritizing of health outcomes; however, recognizing the bi-directional interplay between resource allocation and health outcomes is crucial.
- Evolving political contexts may present challenges in sustaining support for social policy initiatives due to changing political climates.

For peer review only

For peer review only - http://bmjopen.bmj.com/site/about/guidelines.xhtml

INTRODUCTION

Health inequalities are rooted in social structures and systems. Approximately 25% of Dutch citizens find themselves in vulnerable positions (1), meaning that they lack a combination of financial resources, a supportive informal network, a stable living situation, work or daytime activities, education and literacy. Such vulnerabilities can be understood as deprivation in different forms of resources, including economic, social, cultural and personal resources (1). While deprivation may exist in a sole domain, it tends to cumulate across domains (2). All such forms of resources separately have been shown to be related to health outcomes, including years in good health (3), but especially when deprivations cumulate, health loss may be substantial (4). Health loss through adverse lifestyle behaviours (such as problematic alcohol use, smoking and energy-balance-related behaviours) and impaired mental health among individuals in vulnerable positions have been prioritized to reduce health inequalities (5). However, directly targeting mental health and lifestyle behaviours in vulnerable groups has been proven to be problematic, especially at population level, when the underlying deprivations in resources are not addressed (2). Resources such as income and social networks, are thus essential conditions for better mental health and lifestyle behaviours and, relatedly, the reduction of health inequalities.

In welfare states, social policies aim to redistribute resources among citizens, and thereby ameliorate such deprivations of citizens in vulnerable positions. Potentially, they can be tailored towards improving the conditions for health gains in vulnerable groups. However, the impact of social policies, via resources as necessary conditions and mechanisms such as increased agency and reduced stress and burdens, on health outcomes is ill understood. While health benefits are a desired outcome of policies that target vulnerabilities, they are usually not the main target of social policies. Such desired, though unintended, outcomes of policy are best unraveled by understanding social policies as embedded in complex systems with intended and un-intended positive and negative outcomes across a range of levels, domains and actors. This means that social policies can

For peer review only - http://bmjopen.bmj.com/site/about/guidelines.xhtml

sustainably improve but also diminish resources (e.g. financial resources, a supportive social network, employment) for vulnerable groups. Improving health behaviour and mental health are essential for people in vulnerable groups to reduce health inequalities (5). Improving resources is expected to contribute to those necessary improvements, through reducing stress and improving agency.

With this project we target two groups of citizens in vulnerable positions that are subject to social policy: 1) recipients of social assistance benefits, and 2) people with (a risk of) over-indebtedness. In the Netherlands, about 420,000 people receive social assistance (69% > 2 years, 41% > 5 years), while about 650,000 households face problematic debts (6,7). While both groups are primarily defined by deprivation in economic resources, due to the compounding nature, both groups can be expected to also dispose of less other resources. In line with these types of resources being conditions for health status, among both groups, prevalence of (mental) health problems is relatively high (8–10).

To better understand, and ultimately tailor, the way in which social policies contribute to health outcomes for those two groups, municipalities are a strategic point of entry. In the decentralized system of the Netherlands, the national government determines the modalities of social policies such as social assistance benefits and debt policy, while local welfare departments are responsible for processing applications, paying out the monthly benefit, offering employment services, counselling and monitoring claimants, and detecting and sanctioning noncompliance and benefit fraud. There are major differences between municipalities in both the size and composition of the population and causes of vulnerability within the population. In addition, they differ in the available resources for social policy. Therefore, insight is needed in the way in which social policies are embedded in local social policy systems and generate health outcomes.

AIM AND RESEARCH QUESTIONS

SYSTEMS CHANGE TOWARDS SOCIAL POLICIES FOR HEALTH

For peer review only - http://bmjopen.bmj.com/site/about/guidelines.xhtml

With this project, we aim for a systems change in social policies through 1) better understanding how social policy can contribute to reducing health outcomes through improving the redistribution of resources, and 2) implementing those insights to realize conditions necessary to reduce health inequalities. We generate transferable insights that will help the assessment and implementation of social policy interventions that maximize health potential of two vulnerable groups on multiple system levels (i.e. national and local government, professionals, citizens). Through those insights coupled with the development of governance strategies that explicate desired futures, we move from a situation of health impacts as desired but unintended outcomes of social policy, towards a situation where municipalities can better adopt health impact as an intended outcome of their social policies (e.g. a social policies system for health).

To reach this objective, we will answer the following research questions for the aforementioned two groups (i.e. social assistance benefits receivers, and people with (a risk of) problematic debt):

1. What in social policy interventions works to generate health outcomes through the redistribution of resources and burdens, for whom, and under what circumstances?

2. How can the institutional field (i.e. professionals, government) translate insights from RQ1 into strategies to realize conditions to reduce health inequalities?

STATE OF THE ART: SOCIAL POLICY AND HEALTH

In this section, we summarize the state of the art of research on the effects of social policy on health. In general, the underlying mechanisms and transferability of findings to a Dutch-type welfare context often remains unclear. Looking at the Netherlands, there are even substantial differences between regions (urban and rural) and municipalities. In line with the two main target groups for this project, namely people who receive social assistance and people facing problematic debt, we summarize the state of the art of the health impact of two types of social policy that they are subject

For peer review only

to, namely social benefits policy, and debt policy. We tailor our summary towards dimensions of social policies that can be modified in social policy interventions and that point towards opportunities for improved realization of health potential. Strikingly, there is much more known about social assistance policy than debt policy. Research on the Netherlands is scarce in general and there is little to no research that takes an explicit complex systems approach.

Impact of Social Assistance Benefits on Health Outcomes

The literature distinguishes three relevant dimensions of social benefit policies that might affect health outcomes: 1) generosity and eligibility, 2) active labour market policies, and 3) requirements, monitoring and sanctions. Considering 1), in the Netherlands, social assistance is a non-contributory, monthly transfer scheme of last resort for unemployed people who do not or no longer qualify for other benefit schemes, including unemployment benefits. Eligibility for this scheme is based on a work- and means-test, while the benefit level depends on the household composition. In view of 2) labour and reintegration requirements in the Netherlands including active search for a job, willingness to accept all types of paid employment offered, participation in active labour market programs, following education and skill-development programs, signing up with a temporary employment agency (if requested), and performing volunteer work. Lastly 3), as is common to such schemes (11), social assistance claimants have to comply with regulations and obligations, such as administrative requirements (e.g., providing information, attending appointments, and responding to letters and emails) to maintain (full) entitlement to the benefit.

1. Effects of generosity and eligibility of social benefit systems

Findings of a recent literature review show that widened eligibility and increased generosity of social benefit systems are associated with improved mental health and reduced mental health inequalities

For peer review only - http://bmjopen.bmj.com/site/about/guidelines.xhtml

(12). Similarly, reduced eligibility and generosity were associated with deteriorated mental health and increased mental health inequalities.

These latter findings were in line with findings of a literature review examining the health effect of social assistance programs (13). This review shows that stricter eligibility and generosity result in worse health outcomes. Simpson et al. (12) distinguish mechanisms at two levels that might underly these effects: 1) at the aggregate level (e.g., lower generosity and stricter eligibility criteria coincide with stigmatizing attitudes toward benefit claimants and increased income inequality which may subsequently impair mental health) and 2) at the individual level (e.g., increased generosity and eligibility may hamper employment prospects of claimants which subsequently impair mental health). The direction of the effects of expansionary and contractionary policies on mental health is not straightforward as these policies might trigger mechanisms both improving and harming mental health. The underlying mechanisms are complex and might depend on moderating factors.

However, widened eligibility may not improve health in all contexts. In the Netherlands, a randomized experiment among social assistance claimants in The Netherlands shows that imposing a 1-month job search period upon applicants lowered welfare take-up and increased employment rates, while this stricter eligibility regulation did not affect (mental) health (14).

2. Effects of active labor market policies (ALMPs)

A second aspect of social benefits policy that is relevant for health outcomes are Active Labor Market Policies (ALMPs). ALMPs aim to increase re-employment rates and include job search assistance, job or vocational training, and subsidized public and private employment. In general, literature reviews and meta-studies have found that ALMPs have small effects on re-employment in the short run, but larger effects in the medium and long run (e.g., (15,16). Job search assistance programs focusing on 'work first' have similar effects in the short and long run, while job training and

For peer review only - http://bmjopen.bmj.com/site/about/guidelines.xhtml

private sector employment programs increase employment only in the medium and longer run (probably due to lock in effects).

A literature review about the effects of ALMPs on self-reported health outcomes showed that job search assistance including a psychological component (e.g., enhancing self-efficacy) have positive effects on mental health (17). In general, high-risk groups benefitted most. Effects of vocational training programs and subsidized employment were mostly positive but reflected more diversity. In a recent study using administrative data, Caliendo et al. (18) found that participation in a vocational training program reduced drug prescriptions for cardiovascular and mental health problems by about 6-8 percent, while sickness absence reduced by about 20 percent. This effect was larger for vulnerable groups (e.g., lower educated claimants). Effects were likely to be direct (via adoption of a daily routine) rather than indirect (via improved employment prospects) as the reduction in drugs prescription started when claimants were still enrolled in the training program.

Findings in the Netherlands are generally consistent with international evidence. As found in the Dutch social assistance context, entering paid employment has a positive impact on mental and physical health (19). As a consequence, active labor market policies might act as important health promotion policies among unemployed individuals. In the Dutch social assistance context, welfare departments usually provide employment services (including job search assistance and regular encounters with caseworkers) following a target-group based approach. Schuring et al. (20) investigated the effects of different types of employment service approaches for Dutch social assistance claimant using a quasi-experimental approach. They found that Matching (approach focusing on facilitating fast re-employment) reduced drug prescriptions for mental health problems by six percentage points compared to Pre-matching (focus on reducing labor market barriers). These approaches were not significantly different in their impact on other health outcomes (cardiovascular diseases, diabetes, and respiratory illness). Matching also resulted in higher re-employment rates than Pre-matching which might explain the positive finding on mental health. These findings suggest

For peer review only - http://bmjopen.bmj.com/site/about/guidelines.xhtml

that ALMPs could be important to realize a structural breakthrough in improving mental health among vulnerable individuals.

3. Effects of requirements, monitoring, and sanction policy

A third aspect of social benefits policy that can affect health outcomes is the enforcement regime. Economic studies have shown that stricter labor and reintegration requirements, increased monitoring, and more stringent sanction policy tend to decrease unemployment duration and increase job entry rates of (sanctioned) claimants (21–24), although effects often disappear in the long run and these policies might reduce post-unemployment job quality (25–27).

Only a few studies investigated the impact of (elements off) the enforcement regime on health outcomes. Some qualitative and correlational studies conducted in the UK suggest that stricter labour and reintegration requirements, monitoring, and sanction policy are predominantly experienced as stressful and punitive and may negatively affect the mental health and well-being of claimants (28–30). Among claimants of a Danish unemployment benefit program, Baekgaard et al. (31) found that reducing compliance demands to labour and reintegration requirements lowered reported stress levels and increased autonomy. They did not find an effect on stigma. Caliendo et al. (18) investigated the effects of imposed benefit sanctions due to non-compliance to labour and reintegration requirements. They found an increase in drug prescriptions for mental health problems in the month before sanction was imposed (probably due to receiving a warning) and an increase in sickness absence in the months after the sanction was imposed. They did not find a long-run effect on drug prescriptions related to cardiovascular diseases and mental health problems. A potential explanation is that negative health effects of being sanctioned due to financial stress might be compensated by a positive health effect via increased re-employment rates. Understanding the mechanisms in the Dutch context would help to design effective policies with positive health effects both in the short and the long run.

For peer review only - http://bmjopen.bmj.com/site/about/guidelines.xhtml

Impact of Debt Policy on Health Outcomes

In comparison with studies on social assistance benefits policies, studies investigating the effects of debt policy or interventions on health are scarce. We can distinguish three dimensions of debt policy for potential health outcomes: 1) debt counselling, 2) altering the composition of creditors, and 3) debt restructuring and reduction. In the Netherlands, those dimensions are highly interrelated in debt policy. People who are unable to repay their debts on their own can apply for voluntary or statutory debt restructuring based on the law *Wet schuldsanering natuurlijke personen* (Wsnp) (translated into Natural Persons Debt Rehabilitation Act). Municipalities appoint debt counsellors who develop a voluntary payment arrangement based on a calculation of how much the debtor can pay to the creditors each month over a period of three years. If the creditors accept the voluntary payment plan, and the debtor adheres to the plan without entering new debts, the remaining debt is acquitted after three years. If the creditors fail to agree to the payment plan, the citizen can apply for statutory debt restructuring. If the application is granted, the judge appoints an administrator who oversees the process and creditors are obliged to agree with the payment plan. Similar to a voluntary plan, after three years of payments, remaining debts are absolved. Despite this apparent protection offered to people with problematic debts under the law, only a fraction (ca. 14%) of eligible individuals actually seek assistance (32). To make matters worse, the stabilization process that precedes the voluntary repayment plan often takes months and has a high dropout rate. In addition, municipal approaches are often not well tailored to the complex realities of the lives of people with problematic debts, and creditors are not always cooperative. Municipalities differ in the conditions and requirements they impose. For example 20% of the municipalities ask for documentation of the financial situation prior to enrolment, which can be a significant burden for people in problematic debt. It is safe to state that debt policy now fails to reach the large majority of this vulnerable target group, though there are mayor differences between municipalities.

1. Debt counselling

For peer review only - http://bmjopen.bmj.com/site/about/guidelines.xhtml

Of the three dimensions, most evidence is available on the effect of debt counselling on health. A cross-sectional study found that debt counselling significantly reduced financial stressor events among its clients, which in turn was associated with a more positive perception of one's financial situation and perceived health. There were no significant direct effects of debt counselling on perceived health, possible due to the follow-up period of 18 months being too short (33). Similarly, a literature review on the effects of financial counselling shows that there is little evidence for the effect of financial counselling on health. This may be explained by the characteristics of interventions. Most of the interventions that were evaluated put great emphasis on knowledge, while applying behavioural insights might increase the effectiveness of counselling (34). One example of an intervention used in debt counselling that incorporates several behavioural insights is Mobility Mentoring, which was developed by the US-based organization EMPath. Mobility Mentoring has shown promising initial results in the US, but has yet to be proven effective (35). There is scarce evidence in the Netherlands on the effect of debt counselling on client health. A process evaluation of Mobility Mentoring at a Dutch municipality showed that after six months of assistance, clients reported that their health problems were less hindering for their participation in caretaker, educational or work-related activities. One study compared three types of debt counselling In the Netherlands (36). Intensive support across multiple life domains for clients with complex problems, but also target financial counselling combined with administrative and legal support for relatively self-sufficient clients were both suggested to be successful. Success in addressing financial problems coincided with less stress and more belief in a positive outcome in both groups. A counselling type with more limited support accessible for all client types generated less success and less positive health outcomes. Clients experience myriad of emotions, for which there is little attention in the counselling sessions(36). The authors believe that more attention for the client's experience may reduce the attrition rate of debt restructuring trajectories.

2. Altering the composition of creditors

For peer review only - http://bmjopen.bmj.com/site/about/guidelines.xhtml

There is evidence that a smaller number of creditors might have positive health effects. A quasi-experimental study compared households in Singapore that received a certain amount of money to pay off their debts (37). All households received the same amount, which translated into different numbers of creditors that were paid off. Each additional creditor that was paid off was associated with an improved cognitive functioning by a quarter of standard deviation, and 11% less anxiety and 10% less present bias. A qualitative study among UK citizens showed that borrowing from microcredit providers was perceived as having positive effects on health (38). Micro credit reduced stress in the face of unexpected financial costs; it enabled them to maintain social relationships and increased the perceived control over their lives.

3. Debt restructuring and reduction

In a qualitative study on re-employment among participants of debt restructuring programs, participants reported that the start of the voluntary payment plan led to less mental and physical health complaints, such as stress, sleeping problems, and feelings of depression (39).

GAPS IN KNOWLEDGE

While there are some promising leads for reducing health inequalities through social policy, there is still much to be clarified. As discussed, studies investigating the health effects of debt policies are scarce. The literature reviews on social assistance benefit policies report or show a lack of studies conducted in a Dutch-type welfare context.

In addition, there is a lack of studies unravelling the mechanisms underlying the effects of social policies on health outcomes. As a consequence of these gaps, we should be cautious in generalizing the results to the Dutch social assistance benefits context. The present research project aims to fill these gaps for both policy domains. In general, health impacts are usually an unintended by-product of policies that are themselves complex and such effects are embedded and possibly counteracted in

For peer review only - http://bmjopen.bmj.com/site/about/guidelines.xhtml

a complex system (40). In addition, studies usually fail to account for contextual differences (e.g., rural and urban settings) and to address both ex-ante and ex-post effects of social policies. An approach is necessary that combines evidence on impacts, but that also accounts for the way in which contextual variables interact with policy.

METHODS

SYSTEMS APPROACH: REALIST APPROACH AND INSTITUTIONAL APPROACH

We combine two system approaches tailored to the distinct objectives of this project. To generate theory-based-empirically-tested insights in what in social policy works to generate health outcomes, for whom, and under what circumstances, we use a *realist approach*. Firstly, theory based insights are developed in a realist review. Based on the initial theory, together with stakeholders, existing potentially impactful social policy intervention are selected. Secondly, to test these insights empirically, realist evaluations of the selected social policy interventions are conducted. Furthermore, to be able to scale up these insights among institutional actors, we facilitate the development of governance strategies, acknowledging the crucial role of institutional actors to realize a systems change, using an *institutional approach.*

These two system approaches are linked to the two research questions.

REALIST APPROACH

We adopt a realist approach to social policies for four reasons: 1) the focus on mechanisms; 2) the complexity of social policy interventions and systems; 3) the actor perspective, and 4) stakeholder engagement.

For peer review only

Mechanisms

Previously, conventional evaluations of interventions focused on experimental designs, with the RCT as golden standard. This approach has been criticized for its narrowness of scope(41–43), and 'black boxing' effectiveness, especially for complex interventions (44) such as social policy interventions. Alternative approaches have been called for (e.g. (42–45). In this study protocol, we employ such a novel paradigm: the realist approach.

Realist approach is a theory-driven research approach, based on realist philosophy of science (45,46). The underlying assumption is outcomes are produced by mechanisms: the causal power of things (i.e. material things, but also social structures and relationships) to affect other things in specific ways (47). Whether or not the causal power is activated depends on context, for example circumstances (45). The aim is to generate insights in how contexts and mechanisms interact, resulting in outcomes (46,48), thereby unravelling the black box of what works for whom and under what circumstances. This resolves the knowledge gap in current literature (see "state of the art"); it is not well understood how social policy works (i.e. mechanisms), and in what context it works, especially in the Dutch welfare regime. Moreover, insight in the combination of mechanisms and context ensure transferability of the findings. Transferability is particularly relevant, as it allows to develop strategies to roll out or scale up interventions. In this project, these insights are based on theory (realist review) and then empirically tested (realist evaluations).

For peer review only

Complexity

Not only can social policy interventions be considered complex interventions, meaning they contain multiple components, the system in which social policy takes place is also complex. For example, other policies might overlap (i.e. permeable system boundaries), which means that professionals and citizens are not only exposed to the social policy intervention under study. In addition, social

For peer review only - http://bmjopen.bmj.com/site/about/guidelines.xhtml

policy interventions are ‘nested’ in other social systems, like national policies on financing structures and legislation, that influence (the working and the impact) the intervention.

The realist approach allows to study multiple systems levels (and interactions between them), for example:

- on a micro-level: interactions between social policy beneficiaries (i.e. citizens, also called clients in this regard) and professionals/practitioners;
- on the meso-level: organizational issues, such as interorganizational collaboration, or accountability;
- at the macro-level: issues of policy design.

Actor perspective

The aforementioned underlying assumption of realist approach states that human reactions to interventions (i.e. mechanisms) differ according to circumstances (i.e. context) leading to certain outcomes. Different actors on different system levels all have diverse reactions to interventions; social policy intervention not only influence citizens, but also professionals and policymakers. In other words, by employing a realist approach, we provide transferable insights in how different actors respond in a social policy intervention, including what circumstances triggers that reaction (that can for example be national law for policymakers, or overlapping programs for professionals, or previous experiences for citizens).

Stakeholder Involvement

Involvement of stakeholders including the population under study (i.e. people receiving social assistance benefits and people facing overindebtedness) in the research process, is inherent to the realist approach(46).

For peer review only

In the preparation of this study protocol, stakeholders have identified a potentially relevant intervention, and provided input to refine a raw version of an initial program theory (see text box 1). In the project itself, stakeholders will be involved in practical validation of the literature, identifying promising social policy interventions, and in developing the initial program theory, much like in the preparation of the proposal, but more elaborated.

For peer review only

For peer review only - http://bmjopen.bmj.com/site/about/guidelines.xhtml

Text box 1 ILLUSTRATION OF A SOCIAL POLICY INTERVENTION AND ITS INITIAL PROGRAM THEORY

"Vroeg Eropaf" (Early Outreach) is a debt policy intervention designed to address starting problematic debts through a multi-component approach. This illustration provides an overview of the intervention, presents the initial program theory, and outlines the proposed research methodology for testing and refining the theory.

The Vroeg Eropaf intervention aims to identify and address starting problematic debts through three key components. Firstly, a network of service providers signals arrears in payment to municipalities when individuals experience financial difficulties. Secondly, social service teams from municipalities make contact with households when at least two service providers signal arrears, offering assistance if needed. Lastly, if assistance is desired, the teams provide support in managing financial problems by assigning individuals to debt service departments or other societal organizations. This intervention adheres to Dutch and European privacy regulations, and Dutch municipalities have a legal duty to engage in early signaling of debt since January 1st, 2021. Vroeg Eropaf is currently implemented in multiple municipalities, both in urban and rural settings, and has been identified as a potentially relevant intervention for this research.

The initial program theory is informed by existing literature on the impact of overindebtedness on health outcomes. Studies have shown a correlation between debt and various mental health issues, including mental disorders, depression, suicide attempts, problem drinking, drug dependence, neurotic disorders, and psychotic disorders. The underlying mechanisms linking overindebtedness to poor health involve worry, financial scarcity, stress, negative affect, social isolation, reduced perceived control, reduced sleep quality, failure to seek assistance, poor medication adherence, impaired executive functioning, and unhealthy lifestyle behaviors such as unhealthy eating, smoking, and reduced physical activity.

Furthermore, the theory proposes that the reduction of administrative burdens, which individuals may experience in their interactions with the state, plays a significant role in improving health outcomes. Administrative burdens consist of learning costs (searching for information about social programs), compliance costs (meeting administrative requirements), and psychological costs (stress, frustration, loss of autonomy, and stigma). The assistance component of Vroeg Eropaf aims to alleviate these burdens, potentially reducing barriers to accessing debt services and improving overall well-being.

However, it is important to note that the intervention may not have a positive impact on health outcomes for certain groups or under specific conditions. Challenges may arise in effectively reaching all citizens with starting problematic debts, especially if the network of service providers fails to adequately signal or if there are capacity limitations or barriers related to language, literacy, or cultural differences. Additionally, behavioral changes, such as improved financial behavior or increased uptake of debt services, may not occur for some approached individuals due to unproductive interactions with professionals or a lack of trust.

Stakeholder involvement is a crucial aspect of this research, employing 'dual theorizing' by consulting policy advisors and citizens in vulnerable positions. Stakeholders recognize the potential relevance of Vroeg Eropaf for improving health outcomes. Citizens highlighted the interaction between mental health and their response to the intervention, emphasizing that poor mental health can hinder help-seeking behavior. Other stakeholders also acknowledged the significance of administrative burden, considering it a sort of "hygiene condition" that, when present, exacerbates individuals' situations without guaranteeing improvement upon its reduction. Policy advisors expressed an awareness of the burdens placed on citizens and sought alternatives to alleviate them. In a rural municipality, the concept of 'noaberschap' (neighborship), a form of social resource rooted in historical tradition, was identified as potentially impacting the intervention's outcomes by either triggering feelings of shame and hampering health outcomes, or strengthening social resource and improving health outcomes.

For peer review only - http://bmjopen.bmj.com/site/about/guidelines.xhtml

Design and methodology

Realist review

To develop the theoretical insights on what in social policy interventions can generate health outcomes (i.e. improve gains, and prevent losses in terms of lifestyle and mental health), for whom, and under what circumstances, firstly, a realist review is conducted. Informed by these theory-based insights from the realist review, existing relevant social policy interventions will be selected with the municipalities. Initial program theories will be developed for the selected interventions, based on the theory-based insights from the review, combined with stakeholder insights (i.e. “ dual theorizing”, (49).

Realist evaluation

After the realist review, realist evaluation of the selected social policy interventions will be performed. This means that the initial program theories, consisting of multiple context-mechanism-outcome configurations, will be tested, and refined or renewed if necessary. In other words, the theoretical insights from the realist review will be empirically tested, and enriched.

Realist evaluation is method-neutral (46), which entails that methods are chosen on their suitability to empirically test the theory-based context-mechanism-outcome configurations, in order to answer the archetypical realist research question: what works, for whom and under what circumstances. Because of the complexity, (i.e. multiple system levels), a single method may answer only part of the research question. Therefore, we develop tailored multi-method strategies, combining multiple qualitative and/or quantitative methods, according to their contribution to answering the research question.

For peer review only - http://bmjopen.bmj.com/site/about/guidelines.xhtml

As suitability to test the theory-based insights depends on their actual content, and the selection of interventions in the realist synthesis, preceding the realist evaluation phase, we cannot fully define methods a priori. Potentially useful methods are illustrated in text box 2.

Text box 2 ILLUSTRATION OF A MULTI-METHOD EVALUATION STRATEGY

A mixed-method evaluation strategy is utilized to explore various dimensions and levels within the program theory. A combination of document analyses, quasi-experimental design, realist interviews and ethnographic institutional analysis is under consideration for the evaluation of "Vroeg Eropaf" (Early Outreach, see text box 1).

Document analysis entails a meticulous examination of the intervention's implementation and its reach in the municipality under study. This analytical process serves as the foundation for understanding relevant micro, meso, and macro levels and dimensions to be investigated.

We could estimate effects of the Vroeg Eropaf-intervention on resource-related (e.g., labor status and debt situation) and health-related (mental health, physical health) outcomes. To gain insight in the heterogeneity of intervention effects, we will additionally examine whether intervention effects differ among subgroups (e.g., high-risk groups related to (mental) health problems). To identify these intervention effects, we **link administrative data of municipalities to register data of Statistics Netherlands**. Register data of Statistics Netherlands provide individual- and household-level data for all Dutch residents. The data covers several topics ranging from demographics, education, and labour market status to prescribed medication and problematic debts.

Linking municipality data to this data register of Statistics Netherlands provide a unique opportunity to investigate the outcomes of (local) social policy interventions. Recent studies have shown that linking these data sources is feasible and appropriate to identify intervention outcomes of social policy interventions (e.g., 19, 20). To estimate intervention outcomes, we can use quasi-experimental methods, such as instrumental variable approaches, difference-in-difference analyses, regression discontinuity designs, and propensity score matching. These methods fit realist evaluation, as it allows to test the transfactuality of Context-Mechanism-Outcome configurations (60). An appropriate method will be chosen depending on the context characteristics and the availability of data.

Furthermore, on a microlevel, we can investigate the workings of the aforementioned potential mechanisms such as administrative burden, and the contexts that trigger (or prevents them) the mechanisms, such noaberschap in a rural municipality, through **(realist) interviews** with citizens.

Ethnographic institutional analysis of the collaboration between institutional actors allows to explore how micro patterns of professional behavior play a role in the intervention on a mesolevel, and to explore systems-design issues on a macro-level.

Ultimately, this comprehensive combination of methods is aimed at refining, refuting, or revising the context-mechanism-outcome configurations within the program theory, underpinning the workings of the social policy intervention.

Data analysis

Realist review and evaluation analyze literature and data retroductively. Rooted in the belief that comprehending causation requires more than just relying on observable evidence alone, retroduction combines deductive and inductive logic in a back-and-forth-movement, to identify the

potential causal mechanisms that contribute to observed patterns, or variations in those patterns (50). When a program theory is developed in advance, the research process begins with deductive reasoning, which involves seeking evidence to test the theory. Cases are examined, ideally reaching a point of saturation, to ensure that the observed patterns, as well as intended and unintended outcomes, align with the proposed theory. If there are inconsistent cases, it may be necessary to refine the theory. This refinement occurs through the generation of new theory based on observations or inductive reasoning. The newly formulated theory is then tested in additional cases using deductive reasoning once again.

For peer review only

INSTITUTIONAL APPROACH

To develop the aforementioned governance strategies for systems change, we argue that complementary to the realist approach, an institutional approach is needed to understand and change the determining role of institutional actors in the uptake and upscaling of the realist insights.

The institutional approach is an organizational theoretical approach to understanding the interplay between institutions and institutional agents. It views actors as embedded agents, able to develop and transform existing complex systems. Organizational institutionalism focuses on how individuals themselves—as well as through (professional) groups and organizations—are intentional agents of institutional creation, maintenance, and transformation(51).

Design and methodology

The anticipatory governance strategies will be developed in two parts. Firstly, there will be continuous productive interactive meetings with institutional actors throughout the project duration of four years. We draw on theory on collaborative innovation (52) and systemic action research (53) to guide the institutional actors through the collaborative process. The meetings focus on building a

For peer review only - http://bmjopen.bmj.com/site/about/guidelines.xhtml

collaboration process in which institutional actors are invited to analyze the current dynamics of the social policy system, and identify obstructions and opportunities for a social system for health. In addition, the institutional actors explore during the meetings how they, each using their own position and knowledge, can work together with others towards desired changes in the social policy system.

Secondly, a series of workshops will be co-designed using Techniques of Futuring (ToF) (54). Such techniques have been proven successful in realizing system level changes in other domains, for instance in energy transition (55). ToF's can be defined as 'practices bringing together actors around one or more imagined futures and through which actors come to share particular orientations for action' (55) (p225). More specifically, we achieve this by using various established tools, such as 'whole system in the room', stakeholder consultation, and immersive design. See figure 1 for an overview of the series of workshops.

Figure 1 Overview of the co-designed workshops, to develop anticipatory governance strategies from an institutional approach

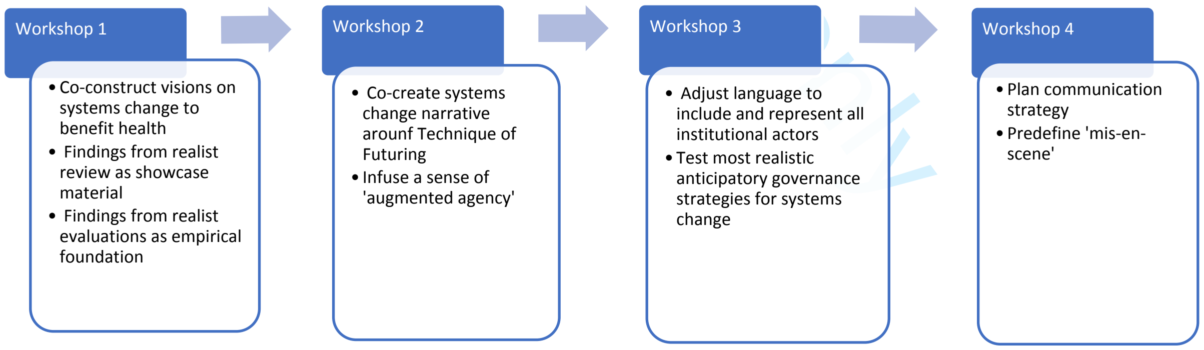

Stakeholder engagement

For peer review only - http://bmjopen.bmj.com/site/about/guidelines.xhtml

Stakeholders involved in the action research are national partners such as professional associations, national government bodies, and interest associations. From the start of the research, they are invited to take an active position in contributing to system change, through their engagement in productive interactive meetings, and reflecting on their own role within the system.

Data analysis

Qualitative data, such as interviews, field notes and recordings, will be analyzed according to thematic content analyses. The analysis focuses on existing and desired institutional dynamics, structures, and logics, mainly expressed through narratives. To ensure quality of analysis, the following measures will be taken into account (56):

- Interpretations will be verified with the institutional partners participating in the action research (member check);

- Multiple data sources will be used and combined in the analyses (data triangulation);

- Data coding will be discussed within the multidisciplinary research team (investigator triangulation);

- Researchers will reflect on their role and influence in the research process (reflexivity);

- Decisions and developments, and the underlying reasons will be documented (audit trail).

OUTCOMES

Priorities for reducing socioeconomic health inequalities identified in the WRR Policy Brief (5) comprise physical activity and diet, smoking, problematic alcohol use (i.e. lifestyle behaviours) and mental health in groups in vulnerable positions. The selected existing social policy interventions are

For peer review only - http://bmjopen.bmj.com/site/about/guidelines.xhtml

evaluated on their impact on outcomes related to these priorities. For example: Statistics Netherlands microdata on antidepressant use for mental health (see text box 2).

TARGET POPULATION

We target two groups with deprivation in (economic) resources: 1) recipients of social assistance benefits (unemployment benefit of last resort), and 2) people with (a risk of) overindebtedness.

SETTINGS

As urban and rural settings might produce differences in vulnerability (i.e. capital) and in governance strategies, having both substantially represented in the study ensures valuable insights for both science and society. In this study, two large municipalities in the strong urbanized West of the Netherlands collaborate. In the more ruralized Mid-East of the Netherlands, 5 municipalities of different sizes (ranging from small to midsize) collaborate.

PATIENT AND PUBLIC INVOLVEMENT STATEMENT

Citizens participate in the study in two ways. Firstly, a participation board of citizens in vulnerable positions participated in the development of the study protocol. They provided research questions and ideas, and input to decide on the selection (criteria) of potential effective interventions. Moreover, they commented on the raw initial program theory of an intervention in a (realist) focus group interview (please see text box 1 for an illustration). Lastly, the participation board will take part in one of the meetings with institutional actors, which focused on the citizens' experiences of the social policy system and its effects on their health.

Secondly, people with mild cognitive limitations are overrepresented among people in vulnerable positions, and so they are an important sub-target group. Therefore, a representative of a national interest group that gives voice to people with cognitive limitations, with lived experiences with

For peer review only - http://bmjopen.bmj.com/site/about/guidelines.xhtml

overindebtedness and receiving social policy interventions will contribute to the research, in for example participating in the development of (communication) materials for citizens.

For peer review only

DISCUSSION

This study protocol has several strengths and potential limitations, which are discussed below., followed by ethics and dissemination.

Strengths and limitations

Strengths

A first strength can be found in the cross-domain systems approach of health inequalities. Many of the various factors influencing health, such as social, economic, environmental, and commercial aspects, which are vital for safeguarding and fostering population health, are in fact not part of the traditional health domain (57). Therefore, the World Health Organization (57) argues to expand the boundaries of what are considered systems that contribute to population health beyond the traditional health systems, into "systems for health". In this perspective, social policy systems are considered to be (potential) systems for health.

A strength of this study protocol is also that it enables both an in-depth understanding of how social policy contributes to health outcomes in citizens in vulnerable positions, and a strategical preparation for uptake and upscaling of these insights. To better understand the mechanisms through which social policy generate health outcomes, and under which contextual circumstances these mechanisms can fire, the project conducts a realist evaluation of existing policy interventions

For peer review only - http://bmjopen.bmj.com/site/about/guidelines.xhtml

embedded in local social policy systems. To strategically prepare for uptake and upscaling of social policy interventions, the project takes an institutional approach to develop such strategies. The combination of these two complementing approaches is both innovative as well as suitable for the required systems change.

Another strength can be found in the settings of this study protocol. Both the forms and causes of vulnerability in populations and social policy practices (including means and possibilities) vary between municipalities, and across rural and urban settings. Both settings are represented in this study by multiple municipalities, varying in size.

A last strength can be found in the transdisciplinarity of this study protocol. To get a full understanding of the workings of social policies, a transdisciplinary team that works in close cooperation is required. The realist approach is particularly appropriate as a catalyst for achieving synergy between disciplines, as it brings together theory in a transdisciplinary way, and methods from different disciplines complement each other. (Behavioral) Health sciences is a multidisciplinary field of study, related to health and healthcare. Especially relevant to the project, is expertise from this field of study on (determinants and psychological pathways of) lifestyle behaviors, but also expertise on evaluating complex interventions. Sociology contributes expertise on the study of inequality in access to diverse forms of resources as well as the contextual nature of social phenomena. Empirical micro-economics adds expertise on micro-economic analyses of behaviour of households, with regard to household finance, employment and health outcomes. Governance and organizational science adds expertise on the development of implementation strategies, drawn from institutional approaches. Furthermore, the realist approach can be considered an integrative approach, as stakeholders are involved throughout the research process, including dual theorizing (i.e. integrating scientific theories with stakeholder theories, (49)), and validation of results. In other words, in this project, scientific knowledge, is combined with professional knowledge, and experiential knowledge.

For peer review only - http://bmjopen.bmj.com/site/about/guidelines.xhtml

Potential limitations

Although the cross-domain approach in this study can be considered promising, health inequalities are a genuine so-called wicked problem. Over the past decennia, despite multiple efforts, the gap in life expectancy between the wealthiest, and the least wealthiest group in the Netherlands, have only grown (5). It can be questioned whether social policy intervention can result in sufficient health gains to genuinely reduce health inequality. Possibly, the potential benefit of social policies lies in protecting the current health status of citizens in vulnerable positions (i.e. no further exacerbation of their health status), rather than actually promoting it (i.e. no actual increase in healthy lifestyle behavior, and improving mental health). However, given the current trend of the ever growing gap, no further exacerbation might then be still considered a relevant step. It might be, that in order to reach health inequality through actual health promotion, not only the social policy domain should be involved, but for example also other policy domains such as the built environment (e.g. the food environment, the physical activity environment, etc.).

Another limitation of the cross-domain approach might be, that some might consider evaluating health impact of policies that have other objectives (such as disposing of sufficient resources) a form of “health imperialism”. However, in this study, the primary objectives of social policies (i.e. redistribution of resources) are considered a crucial part of the pathway to health impact. Health is not considered more important than disposing of sufficient resources, but the bi-directional relation of health and disposing of resources is considered crucial for health inequalities. As such, this study protocol aligns with the health *for* all policies- development (58).

Another potential limitation that can be found in reaching the objective of this study, is the challenge that social policy (i.e. how should burdens and resources be distributed in society, and who is deserving of what?) is subject to political ideology. This particularly might play a role in the action research, for example in the willingness to change. Additionally, the political climate itself

For peer review only - http://bmjopen.bmj.com/site/about/guidelines.xhtml

might change, which, through institutional design, might lead to a challenge for support among stakeholders.

Ethics and dissemination

Ethics

It is important that research participants are not harmed or emotionally burdened. However, data collection directly from citizens has a large added value. Their lived experiences provide relevant and accurate information on contexts, mechanisms and outcomes in the realist evaluation of selected existing social policy interventions. Data may be collected about experiences with deprivation in economic resources, social policies and (mental) health. These can be considered sensitive and stigmatized topics, possibly emotionally burdening them and causing psychological distress.

Where possible, less burdensome forms of data collection will be performed, such as using register data and document analyses. However, in case lived experiences provides indispensable insights, risk of emotional burden for participants will be diminished as much as possible, by properly preparing the methods, for example with mock interviews (59). This will help to get familiar with doing interviews about a certain topic, but also prepare for worst-case scenarios. This way, the researcher can learn to deal with for example psychological distress that can arise as a result of the research questions. The questions will also be prepared to be appropriately sensitive and avoid stigmatizing language (59).

Dissemination

The aim of dissemination within RASP is to reach and promote knowledge uptake among institutional actors at multiple system levels, beyond those involved in the study. The national partners involved in the action research have a large constituency among these actors and effective channels for knowledge dissemination and utilization. Dissemination and utilization activities will therefore align with these channels. In the beginning of the project, a communication plan will be

For peer review only - http://bmjopen.bmj.com/site/about/guidelines.xhtml

formulated that maps out activities of actors involved in the study, that may offer opportunities for knowledge dissemination and utilization. The intended target population will have a key role in determining in which form results will be presented (i.e. co-creation).

We identify six types of relevant target groups for knowledge dissemination. These six target groups are listed below, together with tools, materials and activities, considered relevant for them:

1. Citizens with (risk of) problematic debts and/or receiving social assistance benefits

Communication materials will be developed in co-creation with citizens in vulnerable positions, including a representative from the interest group for people with mild cognitive limitations. An example is an icon folder, or videoclip.

2. Students

Educational materials for students of Social Work, and Social and Legal Services will be developed in co-creation with students, lecturers and current professionals, to ensure fitting of the material in the education program and the daily practice, such as an e-learning module or a guest lecture.

3. Professionals working in public services related to social policy

The publications in professional journals that are planned in this project, target mainly the executing professionals.

4. Policymakers and managers in municipal public services related social policy and/or public health

This target group will be reached through different channels and in different forms; factsheets and infographics will be provided to share the direct insights related to the research, contributions will be made to existing communication channels, such as journals, podcasts and blogs, recurrent symposium. In addition, and an end-symposium will be organized.

5. Policymakers at the national government, working in public health or social policy domain

For peer review only - http://bmjopen.bmj.com/site/about/guidelines.xhtml

This target group will be reached throughout the project as representatives of this group actively take part in the action research component, as well as at the end of the project through an (online) symposium.

6. Scientific community

This community will be reached through scientific publications and presentations at relevant conferences, such as the international debt research group that meets annually at the Law & Society Association Conference.

In addition to the specified target populations, the general audience will be informed about milestones in the project by issuing press releases, and/or LinkedIn messages, and/or corporate communication channels.

Ethics approval and consent to participate

The CCMO, the Dutch Central Commission for Human Research, has assessed this study as not liable to the Medical Research Involving Subjects Act (WMO). This means that it is not required to seek ethical approval by a Medical Ethical Committee. However, ethical approval of research procedures is provided by the Social Sciences ethical committee of Wageningen University. Informed consent to participate in the study is obtained from participants for the use of all forms of personally identifiable data.

Consent for publication

Not applicable

Availability of data and materials

For peer review only - http://bmjopen.bmj.com/site/about/guidelines.xhtml

Not applicable

Competing interests

The authors declare that they have no competing interests.

Funding

This work is supported by the Dutch Research Agenda, executed by NWO and ZonMw, grant number NWA 1333.19.001. Independent peer reviews were part of the application process.

Author Contributions

J.v.B. led the development of the study protocol, and is the research guarantor. J.v.B., E.J.d.B., M.W., Y.M.l.G., E.v.B., H.v.d.H., S.T., M.H., A.H., T.M., M.K., E.d.V. have provided intellectual input and critically reviewed the study protocol. All authors approved the final manuscript.

Acknowledgements

The authors thank the citizens in the advisory board “a healthy future for everyone”, as well as the policy advisors of the collaborating municipalities for their involvement and contributions to the development of this study protocol.

For peer review only

References

1. Vrooman, C., Gijsberts, M., Boelhouwer, J., & Mantext, M. Verschil in Nederland. 2014. (SCP-publicatie).

2. RVS. Gezondheidsverschillen voorbij; Complexe ongelijkheid is een zaak van ons allemaal [Internet]. 2020. Available from: https://adviezen.raadrvs.nl/gezondheidsverschillen-voorbij/

3. Pinxten W, Lievens J. The importance of economic, social and cultural capital in understanding health inequalities: using a Bourdieu-based approach in research on physical and mental health perceptions. Sociol Health Illn. 2014 Sep;36(7):1095–110.

4. Veenstra G, Abel T. Capital interplays and social inequalities in health. Scand J Public Health. 2019 Aug;47(6):631–4.

5. Broeders, D.W.J., Das, H.D., Jennissen, R.P.W., Tiemeijer W.L. & de Visser, M. Van verschil naar potentieel: een realistisch perspectief op de sociaaleconomische gezondheidsverschillen [Internet]. Vol. WRR Policy Brief. 2018. Available from: https://www.wrr.nl/publicaties/policy-briefs/2018/08/27/van-verschil-naar-potentieel.-een-realistisch-perspectief-op-de-sociaaleconomische-gezondheidsverschillen

6. Statistics Netherlands. Personen met een uitkering; uitkeringsontvangers per uitkeringssoort [Internet]. 2021. Available from: https://www.cbs.nl/nl-nl/cijfers/detail/83426NED?q=bijstand

7. Rijksoverheid. Factsheet Participatiewet [Internet]. Ministerie van Sociale Zaken en Werkgelegenheid; 2019. Available from: https://www.rijksoverheid.nl/documenten/brochures/2019/11/26/participatiewet

8. Van Rijnsoever MP, Tromp E, Waterlander WE, Schütz FN, Steenhuis IHM. Verschillen in leefstijl en gezondheid tussen mensen met en zonder schulden. Tijdschr Voor Gezondheidswetenschappen. 2011 Jan;89(1):43–50.

9. Yildiz B, Schuring M, Knoef MG, Burdorf A. Chronic diseases and multimorbidity among unemployed and employed persons in the Netherlands: a register-based cross-sectional study. BMJ Open. 2020 Jul;10(7):e035037.

10. Einerhand, M., & Ravesteijn, B. Psychische klachten en de arbeidsmarkt. 102(4754):481–3.

11. Immervoll,H. K C. How demanding are activation requirements for jobseekers [Internet]. 2018 Jul [cited 2023 Oct 2]. (OECD Social, Employment and Migration Working Papers; vol. 215). Report No.: 215. Available from: https://www.oecd-ilibrary.org/employment/how-demanding-are-activation-requirements-for-jobseekers_2bdfecca-en

12. Simpson J, Albani V, Bell Z, Bambra C, Brown H. Effects of social security policy reforms on mental health and inequalities: A systematic review of observational studies in high-income countries. Soc Sci Med. 2021 Mar;272:113717.

13. Shahidi FV, Ramraj C, Sod-Erdene O, Hildebrand V, Siddiqi A. The impact of social assistance programs on population health: a systematic review of research in high-income countries. BMC Public Health. 2019 Dec;19(1):2.

14. Bolhaar J, Ketel N, Van Der Klaauw B. Job Search Periods for Welfare Applicants: Evidence from a Randomized Experiment. Am Econ J Appl Econ. 2019 Jan 1;11(1):92–125.

For peer review only - http://bmjopen.bmj.com/site/about/guidelines.xhtml

15. Card D, Kluve J, Weber A. What Works? A Meta Analysis of Recent Active Labor Market Program Evaluations. J Eur Econ Assoc. 2018 Jun 1;16(3):894–931.

16. Vooren M, Haelermans C, Groot W, Maassen Van Den Brink H. THE EFFECTIVENESS OF ACTIVE LABOR MARKET POLICIES: A META-ANALYSIS. J Econ Surv. 2019 Feb;33(1):125–49.

17. Puig-Barrachina V, Giró P, Artazcoz L, Bartoll X, Cortés-Franch I, Fernández A, et al. The impact of Active Labour Market Policies on health outcomes: a Scoping review. Eur J Public Health [Internet]. 2019 Mar 25 [cited 2023 Oct 2]; Available from: https://academic.oup.com/eurpub/advance-article/doi/10.1093/eurpub/ckz026/5419277

18. Caliendo M, Mahlstedt R, Van Den Berg GJ, Vikström J. Side Effects of Labor Market Policies. SSRN Electron J [Internet]. 2020 [cited 2023 Oct 2]; Available from: https://www.ssrn.com/abstract=3730444

19. Schuring M, Robroek SJ, Burdorf A. The benefits of paid employment among persons with common mental health problems: evidence for the selection and causation mechanism. Scand J Work Environ Health [Internet]. 2017 Oct 2 [cited 2023 Oct 2]; Available from: http://www.sjweh.fi/show_abstract.php?abstract_id=3675

20. Schuring M, Knoef M, Burdorf A. The effect of employment services on employment and health among unemployed persons: an IV approach. Eur J Public Health. 2022 Oct 21;32(Supplement_3):ckac131.263.

21. Abbring JH, Berg GJ, Ours JC. The Effect of Unemployment Insurance Sanctions on the Transition Rate from Unemployment to Employment. Econ J. 2005 Jul;115(505):602–30.

22. Arni P, Schiprowski A. Job search requirements, effort provision and labor market outcomes. J Public Econ. 2019 Jan;169:65–88.

23. Svarer M. The Effect of Sanctions on Exit from Unemployment: Evidence from Denmark: Effect of Sanctions on Exit from Unemployment. Economica. 2011 Oct;78(312):751–78.

24. Van Den Berg GJ, Van Der Klaauw B, Van Ours JC. Punitive Sanctions and the Transition Rate from Welfare to Work. J Labor Econ. 2004 Jan;22(1):211–41.

25. Arni P, Lalive R, Van Ours JC. HOW EFFECTIVE ARE UNEMPLOYMENT BENEFIT SANCTIONS? LOOKING BEYOND UNEMPLOYMENT EXIT: BENEFIT SANCTIONS BEYOND UNEMPLOYMENT EXIT. J Appl Econom. 2013 Nov;28(7):1153–78.

26. Lachowska M, Meral M, Woodbury SA. Effects of the unemployment insurance work test on long-term employment outcomes. Labour Econ. 2016 Aug;41:246–65.

27. Van Den Berg GJ, Vikström J. Monitoring Job Offer Decisions, Punishments, Exit to Work, and Job Quality. Scand J Econ. 2014 Apr;116(2):284–334.

28. Dwyer P, Scullion L, Jones K, McNeill J, Stewart ABR. Work, welfare, and wellbeing: The impacts of welfare conditionality on people with mental health impairments in the UK. Soc Policy Adm. 2020 Mar;54(2):311–26.

29. Wright S, Fletcher DR, Stewart ABR. Punitive benefit sanctions, welfare conditionality, and the social abuse of unemployed people in Britain: Transforming claimants into offenders? Soc Policy Adm. 2020 Mar;54(2):278–94.

For peer review only - http://bmjopen.bmj.com/site/about/guidelines.xhtml

30. Wright S, Patrick R. Welfare Conditionality in Lived Experience: Aggregating Qualitative Longitudinal Research. Soc Policy Soc. 2019 Oct;18(4):597–613.

31. Baekgaard M, Mikkelsen KS, Madsen JK, Christensen J. Reducing Compliance Demands in Government Benefit Programs Improves the Psychological Well-Being of Target Group Members. J Public Adm Res Theory. 2021 Sep 13;31(4):806–21.

32. Schonewille, G. & Weijers, M. Veel schuldenaren weten schuldhulp slecht te vinden. ESB. 2019;104(4776).

33. Kim, Jinhee and Garman, E. Thomas and Sorhaindo, Benoit,. Relationships Among Credit Counseling Clients' Financial Wellbeing, Financial Behaviors, Financial Stressor Events, and Health [Internet]. Journal of Financial Counseling and Planning; 2003. Available from: https://ssrn.com/abstract=2265623

34. Jungmann, N., Madern, T. Duurzame verandering van financieel gezond gedrag: Droom of werkelijkheid? [Internet]. WRR; 2016. Available from: https://www.wrr.nl/publicaties/working-papers/2016/06/30/duurzame-verbetering-van-gezond-financieel-gedrag.-droom-of-werkelijkheid#:~:text=Droom%20of%20werkelijkheid%3F%20is%20uitgevoerd,gezonder%20financieel%20gedrag%20gaan%20vertonen.

35. Washington State Department of Early Learning. Year one report - ECEAP Family Support Pilot june 2015 -june 2016 [Internet]. Olympia; 2016. Available from: https://dcyf.wa.gov/sites/default/files/pdf/eceap/ECEAP_Family_Support_Pilot_Report_Final.pdf

36. van Logtestijn, B. van der Want,E. van Jeveren, F., Dirven, J. & Broere, M. Gemeenten zetten de schouders eronder [Internet]. 2019. Available from: https://vng.nl/publicaties/gemeenten-zetten-de-schouders-eronder

37. Ong Q, Theseira W, Ng IYH. Reducing debt improves psychological functioning and changes decision-making in the poor. Proc Natl Acad Sci. 2019 Apr 9;116(15):7244–9.

38. Myrskylä M, Gagnon A, Bengtsson T. Pathways to Health and Well-being. Soc Sci Med. 2014 Oct;119:175–9.

39. van der Laan, J. & van Geuns, R. Schuldenvrij: De weg naar werk? [Internet]. 2016. Available from: https://www.hva.nl/kmr/gedeelde-content/publicaties/publicaties-algemeen/2016/schuldenvrij-de-weg-naar-werk.html

40. Shiell A, Hawe P, Gold L. Complex interventions or complex systems? Implications for health economic evaluation. BMJ. 2008 Jun 7;336(7656):1281–3.

41. Cartwright N. Are RCTs the Gold Standard? BioSocieties. 2007 Mar;2(1):11–20.

42. Craig P, Dieppe P, Macintyre S, Michie S, Nazareth I, Petticrew M. Developing and evaluating complex interventions: the new Medical Research Council guidance. BMJ. 2008 Sep 29;a1655.

43. Craig P, Dieppe P, Macintyre S, Michie S, Nazareth I, Petticrew M. Developing and evaluating complex interventions: The new Medical Research Council guidance. Int J Nurs Stud. 2013 May;50(5):587–92.

For peer review only - http://bmjopen.bmj.com/site/about/guidelines.xhtml

44. Larouche A, Potvin L. Stimulating innovative research in health promotion. Glob Health Promot. 2013 Jun;20(2):64–9.

45. Wong G, Westhorp G, Manzano A, Greenhalgh J, Jagosh J, Greenhalgh T. RAMESES II reporting standards for realist evaluations. BMC Med. 2016 Dec;14(1):96.

46. Pawson, R., & Tilley, N. Realistic evaluation. SAGE Publications Ltd; 1997.

47. Westhorp G. Understanding Mechanisms in Realist Evaluation and Research. In: Doing Realist Research [Internet]. 1 Oliver's Yard, 55 City Road London EC1Y 1SP: SAGE Publications Ltd; 2018 [cited 2023 Oct 2]. p. 41–58. Available from: https://methods.sagepub.com/book/doing-realist-research/i249.xml

48. Salter KL, Kothari A. Using realist evaluation to open the black box of knowledge translation: a state-of-the-art review. Implement Sci. 2014 Dec;9(1):115.

49. Emmel N, Greenhalgh J, Manzano A, Monaghan M, Dalkin S. Doing Realist Research [Internet]. 1 Oliver's Yard, 55 City Road London EC1Y 1SP: SAGE Publications Ltd; 2018 [cited 2023 Oct 2]. Available from: https://methods.sagepub.com/book/doing-realist-research

50. Jagosh J. Retroductive theorizing in Pawson and Tilley's applied scientific realism. J Crit Realism. 2020 Mar 14;19(2):121–30.

51. Greenwood R, Oliver C, Lawrence T, Meyer R. The SAGE Handbook of Organizational Institutionalism [Internet]. 1 Oliver's Yard, 55 City Road London EC1Y 1SP: SAGE Publications Ltd; 2017 [cited 2023 Oct 2]. Available from: https://sk.sagepub.com/reference/sage-handbook-of-organizational-institutionalism-2e

52. Lopes AV, Farias JS. How can governance support collaborative innovation in the public sector? A systematic review of the literature. Int Rev Adm Sci. 2022 Mar;88(1):114–30.

53. Burns D. Systemic Action Research: A Strategy for Whole System Change [Internet]. 1st ed. Bristol University Press; 2007 [cited 2023 Oct 2]. Available from: https://www.cambridge.org/core/product/identifier/9781847422750/type/book

54. Oomen J, Hoffman J, Hajer MA. Techniques of futuring: On how imagined futures become socially performative. Eur J Soc Theory. 2022 May;25(2):252–70.

55. Hajer MA, Pelzer P. 2050—An Energetic Odyssey: Understanding 'Techniques of Futuring' in the transition towards renewable energy. Energy Res Soc Sci. 2018 Oct;44:222–31.

56. Frambach JM, van der Vleuten CP, Durning SJ. AM Last Page: Quality Criteria in Qualitative and Quantitative Research. 2013;88(4):552.

57. World Health Organization, Shroff ZC, Marten R, Hanson K. Systems for health: everyone has a role: flagship report of the Alliance for Health Policy and Systems Research [Internet]. Geneva: World Health Organization; 2022. Available from: https://iris.who.int/handle/10665/363923

58. Greer SL, Falkenbach M, Siciliani L, McKee M, Wismar M, Figueras J. From Health in All Policies to Health for All Policies. Lancet Public Health. 2022 Aug;7(8):e718–20.

59. Melville, A. H D. Conducting Sensitive Interviews: A Review of Reflections. Law Method. 2016. 10.5553/REM/.000015

For peer review only - http://bmjopen.bmj.com/site/about/guidelines.xhtml

Figure 1 Overview of the co-designed workshops, to develop anticipatory governance strategies from an institutional approach

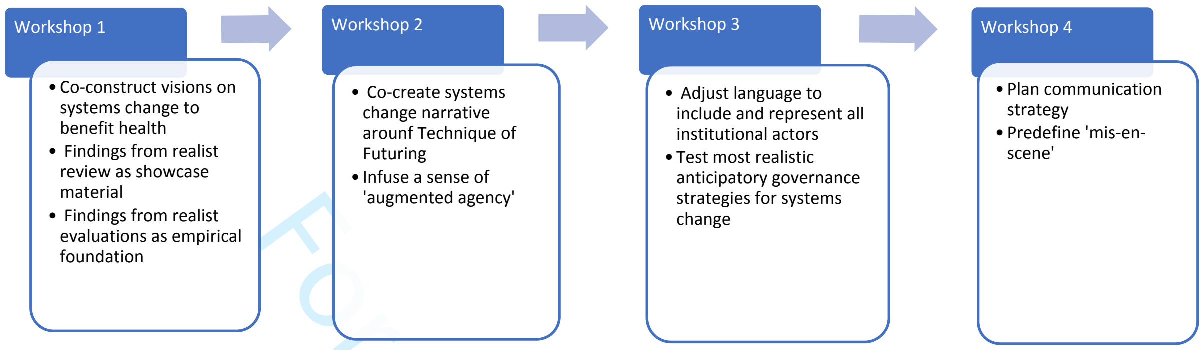

For peer review only

Text box 1 ILLUSTRATION OF A SOCIAL POLICY INTERVENTION AND ITS INITIAL PROGRAM THEORY

"Vroeg Eropaf" (Early Outreach) is a debt policy intervention designed to address starting problematic debts through a multi-component approach. This illustration provides an overview of the intervention, presents the initial program theory, and outlines the proposed research methodology for testing and refining the theory.

The Vroeg Eropaf intervention aims to identify and address starting problematic debts through three key components. Firstly, a network of service providers signals arrears in payment to municipalities when individuals experience financial difficulties. Secondly, social service teams from municipalities make contact with households when at least two service providers signal arrears, offering assistance if needed. Lastly, if assistance is desired, the teams provide support in managing financial problems by assigning individuals to debt service departments or other societal organizations. This intervention adheres to Dutch and European privacy regulations, and Dutch municipalities have a legal duty to engage in early signaling of debt since January 1st, 2021. Vroeg Erepaf is currently implemented in multiple municipalities, both in urban and rural settings, and has been identified as a potentially relevant intervention for this research.

The initial program theory is informed by existing literature on the impact of overindebtedness on health outcomes. Studies have shown a correlation between debt and various mental health issues, including mental disorders, depression, suicide attempts, problem drinking, drug dependence, neurotic disorders, and psychotic disorders. The underlying mechanisms linking overindebtedness to poor health involve worry, financial scarcity, stress, negative affect, social isolation, reduced perceived control, reduced sleep quality, failure to seek assistance, poor medication adherence, impaired executive functioning, and unhealthy lifestyle behaviors such as unhealthy eating, smoking, and reduced physical activity.

Furthermore, the theory proposes that the reduction of administrative burdens, which individuals may experience in their interactions with the state, plays a significant role in improving health outcomes. Administrative burdens consist of learning costs (searching for information about social programs), compliance costs (meeting administrative requirements), and psychological costs (stress, frustration, loss of autonomy, and stigma). The assistance component of Vroeg Eropaf aims to alleviate these burdens, potentially reducing barriers to accessing debt services and improving overall well-being.

However, it is important to note that the intervention may not have a positive impact on health outcomes for certain groups or under specific conditions. Challenges may arise in effectively reaching all citizens with starting problematic debts, especially if the network of service providers fails to adequately signal or if there are capacity limitations or barriers related to language, literacy, or cultural differences. Additionally, behavioral changes, such as improved financial behavior or increased uptake of debt services, may not occur for some approached individuals due to unproductive interactions with professionals or a lack of trust.

Stakeholder involvement is a crucial aspect of this research, employing 'dual theorizing' by consulting policy advisors and citizens in vulnerable positions. Stakeholders recognize the potential relevance of Vroeg Eropaf for improving health outcomes. Citizens highlighted the interaction between mental health and their response to the intervention, emphasizing that poor mental health can hinder help-seeking behavior. Other stakeholders also acknowledged the significance of administrative burden, considering it a sort of "hygiene condition" that, when present, exacerbates individuals' situations without guaranteeing improvement upon its reduction. Policy advisors expressed an awareness of the burdens placed on citizens and sought alternatives to alleviate them. In a rural municipality, the concept of 'noaberschap' (neighborship), a form of social resource rooted in historical tradition, was identified as potentially impacting the intervention's outcomes by either triggering feelings of shame and hampering health outcomes, or strengthening social resource and improving health outcomes.

Text box 2 ILLUSTRATION OF A MULTI-METHOD EVALUATION STRATEGY

A mixed-method evaluation strategy is utilized to explore various dimensions and levels within the program theory. A combination of document analyses, quasi-experimental design, realist interviews and ethnographic institutional analysis is under consideration for the evaluation of “Vroeg Eropaf” (Early Outreach, see text box 1).

Document analysis entails a meticulous examination of the intervention's implementation and its reach in the municipality under study. This analytical process serves as the foundation for understanding relevant micro, meso, and macro levels and dimensions to be investigated.

We could estimate effects of the Vroeg Eropaf-intervention on resource-related (e.g., labor status and debt situation) and health-related (mental health, physical health) outcomes. To gain insight in the heterogeneity of intervention effects, we will additionally examine whether intervention effects differ among subgroups (e.g., high-risk groups related to (mental) health problems). To identify these intervention effects, we **link administrative data of municipalities to register data of Statistics Netherlands**. Register data of Statistics Netherlands provide individual- and household-level data for all Dutch residents. The data covers several topics ranging from demographics, education, and labour market status to prescribed medication and problematic debts.

Linking municipality data to this data register of Statistics Netherlands provide a unique opportunity to investigate the outcomes of (local) social policy interventions. Recent studies have shown that linking these data sources is feasible and appropriate to identify intervention outcomes of social policy interventions (e.g., 19, 20). To estimate intervention outcomes, we can use quasi-experimental methods, such as instrumental variable approaches, difference-in-difference analyses, regression discontinuity designs, and propensity score matching. These methods fit realist evaluation, as it allows to test the transfactuality of Context-Mechanism-Outcome configurations (60). An appropriate method will be chosen depending on the context characteristics and the availability of data.

Furthermore, on a microlevel, we can investigate the workings of the aforementioned potential mechanisms such as administrative burden, and the contexts that trigger (or prevents them) the mechanisms, such noaberschap in a rural municipality, through **(realist) interviews** with citizens.

Ethnographic institutional analysis of the collaboration between institutional actors allows to explore how micro patterns of professional behavior play a role in the intervention on a mesolevel, and to explore systems-design issues on a macro-level.

Ultimately, this comprehensive combination of methods is aimed at refining, refuting, or revising the context-mechanism-outcome configurations within the program theory, underpinning the workings of the social policy intervention.

nly

BMJ Open

The Realist Approach to Social Policies (RASP) study to reduce socioeconomic health inequalities through systems change: protocol for a research project combining mixed-methods realist research with institutional action research

Journal:	*BMJ Open*
Manuscript ID	bmjopen-2024-088571.R1
Article Type:	Protocol
Date Submitted by the Author:	28-May-2024
Complete List of Authors:	Van Berkel, Jantien; Utrecht University, Interdisciplinary Social Science: Public Health; Wageningen Universiteit en Research, Consumption and Healthy Lifestyles<br>de Bruijn, Ernst-Jan; Leiden University, Institute of Tax Law and Economics | Department of Economics<br>Waardenburg, Maikel; Utrecht University, Law, Economics and Governance; Utrecht University School of Governance<br>la Grouw, Yvonne; Utrecht University, Law, Economics and Governance; Utrecht University School of Governance<br>van Bennekom, Eline; Wageningen Universiteit en Research, Consumption and Healthy Lifestyles; Utrecht University, Interdisciplinary Social Science: Public Health<br>van der Horst, Hilje; Wageningen Universiteit en Research, Consumption and Healthy Lifestyles<br>Tonnon, Susanne; Hogeschool Utrecht, Debt and Collection<br>Haggenburg- Mohammed, Milou; Gemeente Amsterdam, Onderzoek en Statistiek<br>Haveman-Nies, Annemien; Wageningen Universiteit en Research, Consumption and Healthy Lifestyles; GGD Noord en Oost Gelderland, AGORA Academische werkplaats<br>Madern, Tamara; Hogeschool Utrecht, Debt and Collection<br>Knoef, Marike; Tilburg University, Tilburg School of Economics and Management; Leiden University, Institute of Tax Law and Economics | Department of Economics<br>de Vet, Emely; Tilburg University, Tilburg School of Humanities and Digital Sciences; Liberal Arts and Sciences; Wageningen Universiteit en Research, Consumption and Healthy Lifestyles
**Primary Subject Heading**:	Public health
Secondary Subject Heading:	Sociology
Keywords:	Health Equity, Community-Based Participatory Research, MENTAL HEALTH, Overweight, Smoking Reduction

For peer review only - http://bmjopen.bmj.com/site/about/guidelines.xhtml

The Realist Approach to Social Policies (RASP) study to reduce socioeconomic health inequalities through systems change: protocol for a research project combining mixed-methods realist research with institutional action research

Authors, email addresses and affiliated institutions:

Jantien van Berkel, j.vanberkel@uu.nl [1,2]

Ernst-Jan de Bruijn, e.de.bruijn@law.leidenuniv.nl [3]

Maikel Waardenburg, M.Waardenburg@uu.nl [4]

Yvonne la Grouw, y.m.lagrouw@uu.nl [4]

Eline van Bennekom, eline.vanbennekom@wur.nl [2,1]

Hilje van der Horst, Hilje.vanderhorst@wur.nl [2]

Susanne Tonnon, susanne.tonnon@hu.nl [5]

Milou Haggenburg- Mohammed, m.haggenburg@amsterdam.nl [6]

Annemien Haveman, annemien.haveman@wur.nl [2,7]

Tamara Madern, tamara.madern@hu.nl [5]

Marike Knoef, M.G.Knoef@tilburguniversity.edu [8]

Emely de Vet, E.W.M.L.deVet@tilburguniversity.edu [9,2]

1 Utrecht University | Department of Interdisciplinary Social Science | Padualaan 14, 3584 CH Utrecht |Netherlands

For peer review only - http://bmjopen.bmj.com/site/about/guidelines.xhtml

2 Wageningen University and Research | Chairgroup Consumption and Healthy Lifestyles | Hollandseweg 1, 6706 KN Wageningen | Netherlands

3 Leiden University | Department of Economics| Steenschuur 25, 2311 ES Leiden | Netherlands

4 Utrecht University| School of Governance | Bijlhouwerstraat 6-8, 3511 ZC Utrecht | Netherlands

5 University of Applied Sciences Utrecht | Research Group Debt and Collection| Padualaan 101, 3584 CH Utrecht | Netherlands

6 Gemeente Amsterdam | Department of Research and Statistics | Oudezijds Voorburgwal 300, 1012 GL Amsterdam| Netherlands

7 GGD-Noord- en Oost Gelderland| Rijksstraatweg 65, 7231 AC Warnsveld| Netherlands

8 Tilburg University | Tilburg School of Economics and Management | PO Box 90153, 5000 LE Tilburg| Netherlands

9 Tilburg University| Tilburg School of Humanities and Digital Sciences | PO Box 90153, 5000 LE Tilburg | Netherlands

Correspondence to:

Jantien van Berkel

PO Box 80140, 3508 TC, Utrecht, Netherlands

E-mail: j.vanberkel@uu.nl

Telephone: 06-48115026

Keywords: Health Equity; Community-Based Participatory Research; MENTAL HEALTH; Overweight; Smoking Reduction

Word count: 4475 words

For peer review only

Abstract

Introduction

Health inequalities are rooted in inequality in vital resources for health, including financial resources, a supportive informal network, a stable living situation, work or daytime activities, or education and literacy. About 25% of Dutch citizens experience deprivation in such resources. Social policy consists of crucial instruments for improving resources in those groups, but can also have adverse effects and lead to additional burden. This project aims to contribute to the reduction of health inequalities through 1) better understanding how social policy interventions can contribute to reducing health inequality through the redistribution of burdens and resources, and 2) developing anticipatory governance strategies to implement those insights, contributing to a change in social policy systems.

Methods and analysis

Two systems approaches are combined for establishing a systems change in the Netherlands. Firstly, a realist approach enables insights into what in social policy interventions may impact health outcomes, for whom, and under what circumstances. Secondly, an institutional approach enables scaling up these insights, by acknowledging the crucial role of institutional actors for accomplishing a systems change. Together with stakeholders, we perform a realist review of the literature and identify existing promising social policy interventions. Next, we execute mixed-methods realist evaluations of selected social policy interventions in seven municipalities, ranging from small, mid-size to large, and in both urban and rural settings. Simultaneously, through action research with (national) institutional actors, we facilitate development of anticipatory governance strategies.

Ethics and dissemination

This study is not liable to the Medical Research Involving Subjects Act (WMO). Informed consent to participate in the study is obtained from participants for the use of all forms of personally identifiable data. Dissemination will be co-developed with target populations, and includes

For peer review only - http://bmjopen.bmj.com/site/about/guidelines.xhtml

communication materials for citizens, education materials for students, workshops, infographics and decision tools for policymakers, and publications for professionals.

For peer review only

Strengths and limitations of this study

- The cross-domain systems approach expands understanding beyond traditional health systems by integrating social policy as potential systems for health.
- Realist evaluation provides insight into how social policy contributes to health outcomes and prepares for strategic uptake and upscaling.
- Diverse settings are represented, including both urban and rural municipalities, capturing variations in vulnerability and social policy practices.
- The "health imperialism" critique suggests that evaluating policies primarily focused on resource distribution may raise ethical concerns about the prioritizing of health outcomes; however, recognizing the bi-directional interplay between resource allocation and health outcomes is crucial.
- Evolving political contexts may present challenges in sustaining support for social policy initiatives due to changing political climates.

For peer review only

INTRODUCTION

Health inequalities are rooted in social structures and systems. Approximately 25% of Dutch citizens find themselves in vulnerable positions (1), meaning that they lack a combination of financial resources, a supportive informal network, a stable living situation, work or daytime activities, education and literacy. Such vulnerabilities can be understood as deprivation in different forms of resources, including economic, social, cultural and personal resources (1). While deprivation may exist in a sole domain, it tends to cumulate across domains (2). All such forms of resources separately have been shown to be related to health outcomes, including years in good health (3), but especially when deprivations cumulate, health loss may be substantial (4). Health loss through adverse lifestyle behaviours (such as problematic alcohol use, smoking and energy-balance-related behaviours) and impaired mental health among individuals in vulnerable positions have been prioritized to reduce health inequalities (5). However, directly targeting mental health and lifestyle behaviours in vulnerable groups has been proven to be problematic, especially at population level, when the underlying deprivations in resources are not addressed (2). Resources such as income and social networks, are thus essential conditions for better mental health and lifestyle behaviours and, relatedly, the reduction of health inequalities.

In welfare states, social policies aim to redistribute resources among citizens, and thereby ameliorate such deprivations of citizens in vulnerable positions. Potentially, they can be tailored towards improving the conditions for health gains in vulnerable groups. However, the impact of social policies, via resources as necessary conditions and mechanisms such as increased agency and reduced stress and burdens, on health outcomes is ill understood. While health benefits are a desired outcome of policies that target vulnerabilities, they are usually not the main target of social policies. Such desired, though unintended, outcomes of policy are best unraveled by understanding social policies as embedded in complex systems with intended and un-intended positive and negative outcomes across a range of levels, domains and actors. This means that social policies can

For peer review only - http://bmjopen.bmj.com/site/about/guidelines.xhtml

sustainably improve but also diminish resources (e.g. financial resources, a supportive social network, employment) for vulnerable groups. Improving health behaviour and mental health are essential for people in vulnerable groups to reduce health inequalities (5). Improving resources is expected to contribute to those necessary improvements, through reducing stress and improving agency.

With this project we target two groups of citizens in vulnerable positions that are subject to social policy: 1) recipients of social assistance benefits, and 2) people with (a risk of) over-indebtedness. In the Netherlands, about 420,000 people receive social assistance (69% > 2 years, 41% > 5 years), while about 650,000 households face problematic debts (6,7). While both groups are primarily defined by deprivation in economic resources, due to the compounding nature, both groups can be expected to also dispose of less other resources. In line with these types of resources being conditions for health status, among both groups, prevalence of (mental) health problems is relatively high (8–10). An extensive summary of the state of the art of research into the effects of social assistance benefits policy and debt policy on the health of their target populations can be found in **supplemental file 1**.

To better understand, and ultimately tailor, the way in which social policies contribute to health outcomes for those two groups, municipalities are a strategic point of entry. In the decentralized system of the Netherlands, the national government determines the modalities of social policies such as social assistance benefits and debt policy, while local welfare departments are responsible for processing applications, paying out the monthly benefit, offering employment services, counselling and monitoring claimants, and detecting and sanctioning noncompliance and benefit fraud. There are major differences between municipalities in both the size and composition of the population and causes of vulnerability within the population. In addition, they differ in the available resources for social policy. Therefore, insight is needed in the way in which social policies are embedded in local social policy systems and generate health outcomes.

For peer review only - http://bmjopen.bmj.com/site/about/guidelines.xhtml

Aim and research questions

Systems change towards social policies for health

With this project, we aim for a systems change in social policies through 1) better understanding how social policy can contribute to reducing health outcomes through improving the redistribution of resources, and 2) implementing those insights to realize conditions necessary to reduce health inequalities. We generate transferable insights that will help the assessment and implementation of social policy interventions that maximize health potential of two vulnerable groups on multiple system levels (i.e. national and local government, professionals, citizens). Through those insights coupled with the development of governance strategies that explicate desired futures, we move from a situation of health impacts as desired but unintended outcomes of social policy, towards a situation where municipalities can better adopt health impact as an intended outcome of their social policies (e.g. a social policies system for health).

To reach this objective, we will answer the following research questions for the aforementioned two groups (i.e. social assistance benefits receivers, and people with (a risk of) problematic debt):

1. What in social policy interventions works to generate health outcomes through the redistribution of resources and burdens, for whom, and under what circumstances?

2. How can the institutional field (i.e. professionals, government) translate insights from RQ1 into strategies to realize conditions to reduce health inequalities?

METHODS AND ANALYSIS

Systems approach: realist approach and institutional approach

We combine two system approaches tailored to the distinct objectives of this project. To generate theory-based-empirically-tested insights in what in social policy works to generate health outcomes,

For peer review only - http://bmjopen.bmj.com/site/about/guidelines.xhtml

for whom, and under what circumstances, we use a *realist approach*. Firstly, theory based insights are developed in a realist review. Based on the initial theory, together with stakeholders, existing potentially impactful social policy intervention are selected. Secondly, to test these insights empirically, realist evaluations of the selected social policy interventions are conducted. Furthermore, to be able to scale up these insights among institutional actors, we facilitate the development of governance strategies, acknowledging the crucial role of institutional actors to realize a systems change, using an *institutional approach.*

These two system approaches are linked to the two research questions.

Realist approach

We adopt a realist approach to social policies for four reasons: 1) the focus on mechanisms; 2) the complexity of social policy interventions and systems; 3) the actor perspective, and 4) stakeholder engagement.

Mechanisms

Previously, conventional evaluations of interventions focused on experimental designs, with the RCT as golden standard. This approach has been criticized for its narrowness of scope (11–13), and 'black boxing' effectiveness, especially for complex interventions (14) such as social policy interventions. Alternative approaches have been called for (e.g. (12–15). In this study protocol, we employ such a novel paradigm: the realist approach.

Realist approach is a theory-driven research approach, based on realist philosophy of science (15,16). The underlying assumption is outcomes are produced by mechanisms: the causal power of things (i.e. material things, but also social structures and relationships) to affect other things in specific ways (17). Whether or not the causal power is activated depends on context, for example

For peer review only - http://bmjopen.bmj.com/site/about/guidelines.xhtml

circumstances (15). The aim is to generate insights in how contexts and mechanisms interact, resulting in outcomes (16,18), thereby unravelling the black box of what works for whom and under what circumstances. This resolves the knowledge gap in current literature (see “state of the art”); it is not well understood how social policy works (i.e. mechanisms), and in what context it works, especially in the Dutch welfare regime. Moreover, insight in the combination of mechanisms and context ensure transferability of the findings. Transferability is particularly relevant, as it allows to develop strategies to roll out or scale up interventions. In this project, these insights are based on theory (realist review) and then empirically tested (realist evaluations).

Complexity

Not only can social policy interventions be considered complex interventions, meaning they contain multiple components, the system in which social policy takes place is also complex. For example, other policies might overlap (i.e. permeable system boundaries), which means that professionals and citizens are not only exposed to the social policy intervention under study. In addition, social policy interventions are ‘nested’ in other social systems, like national policies on financing structures and legislation, that influence (the working and the impact) the intervention.

The realist approach allows to study multiple systems levels (and interactions between them), for example:

- on a micro-level: interactions between social policy beneficiaries (i.e. citizens, also called clients in this regard) and professionals/practitioners;
- on the meso-level: organizational issues, such as interorganizational collaboration, or accountability;
- at the macro-level: issues of policy design.

For peer review only

Actor perspective

The aforementioned underlying assumption of realist approach states that human reactions to interventions (i.e. mechanisms) differ according to circumstances (i.e. context) leading to certain outcomes. Different actors on different system levels all have diverse reactions to interventions; social policy intervention not only influence citizens, but also professionals and policymakers. In other words, by employing a realist approach, we provide transferable insights in how different actors respond in a social policy intervention, including what circumstances triggers that reaction (that can for example be national law for policymakers, or overlapping programs for professionals, or previous experiences for citizens).

Stakeholder involvement

Involvement of stakeholders including the population under study (i.e. people receiving social assistance benefits and people facing overindebtedness) in the research process, is inherent to the realist approach (16).

In the preparation of this study protocol, stakeholders have identified a potentially relevant intervention, and provided input to refine a raw version of an initial program theory (see **supplemental text box 1**). In the project itself, stakeholders will be involved in practical validation of the literature, identifying promising social policy interventions, and in developing the initial program theory, much like in the preparation of the proposal, but more elaborated.

Design and methodology

Realist review

To develop the theoretical insights on what in social policy interventions can generate health outcomes (i.e. improve gains, and prevent losses in terms of lifestyle and mental health), for whom,

For peer review only - http://bmjopen.bmj.com/site/about/guidelines.xhtml

and under what circumstances, firstly, a realist review is conducted. Informed by these theory-based insights from the realist review, existing relevant social policy interventions will be selected with the municipalities. Initial program theories will be developed for the selected interventions, based on the theory-based insights from the review, combined with stakeholder insights (i.e. “ dual theorizing”, (19).

Realist evaluation

After the realist review, realist evaluation of the selected social policy interventions will be performed. This means that the initial program theories, consisting of multiple context-mechanism-outcome configurations, will be tested, and refined or renewed if necessary. In other words, the theoretical insights from the realist review will be empirically tested, and enriched.

Realist evaluation is method-neutral (16), which entails that methods are chosen on their suitability to empirically test the theory-based context-mechanism-outcome configurations, in order to answer the archetypical realist research question: what works, for whom and under what circumstances. Because of the complexity, (i.e. multiple system levels), a single method may answer only part of the research question. Therefore, we develop tailored multi-method strategies, combining multiple qualitative and/or quantitative methods, according to their contribution to answering the research question.

As suitability to test the theory-based insights depends on their actual content, and the selection of interventions in the realist synthesis, preceding the realist evaluation phase, we cannot fully define methods a priori. Potentially useful methods are illustrated in **supplemental text box 2**.

Data analysis

Realist review and evaluation analyze literature and data retroductively. Rooted in the belief that comprehending causation requires more than just relying on observable evidence alone,

For peer review only - http://bmjopen.bmj.com/site/about/guidelines.xhtml

For peer review only

retroduction combines deductive and inductive logic in a back-and-forth-movement, to identify the potential causal mechanisms that contribute to observed patterns, or variations in those patterns (20). When a program theory is developed in advance, the research process begins with deductive reasoning, which involves seeking evidence to test the theory. Cases are examined, ideally reaching a point of saturation, to ensure that the observed patterns, as well as intended and unintended outcomes, align with the proposed theory. If there are inconsistent cases, it may be necessary to refine the theory. This refinement occurs through the generation of new theory based on observations or inductive reasoning. The newly formulated theory is then tested in additional cases using deductive reasoning once again.

Realist review is planned from September 2022 until December 2023. Preparations for data collection for realist evaluation (i.e. stakeholders involved in decision processes) start in September 2023, actual start of data collection (i.e. inclusion of participants) is in September 2024. Results of the review can be expected in 2024. Results of the evaluations can be expected in 2025, 2026 and 2027.

Institutional approach

To develop the aforementioned governance strategies for systems change, we argue that complementary to the realist approach, an institutional approach is needed to understand and change the determining role of institutional actors in the uptake and upscaling of the realist insights.

The institutional approach is an organizational theoretical approach to understanding the interplay between institutions and institutional agents. It views actors as embedded agents, able to develop and transform existing complex systems. Organizational institutionalism focuses on how individuals themselves—as well as through (professional) groups and organizations—are intentional agents of institutional creation, maintenance, and transformation (21).

For peer review only

For peer review only - http://bmjopen.bmj.com/site/about/guidelines.xhtml

Design and methodology

The anticipatory governance strategies will be developed in two parts. Firstly, there will be continuous productive interactive meetings with institutional actors throughout the project duration of four years. We draw on theory on collaborative innovation (22) and systemic action research (23) to guide the institutional actors through the collaborative process. The meetings focus on building a collaboration process in which institutional actors are invited to analyse the current dynamics of the social policy system, and identify obstructions and opportunities for a social system for health. In addition, the institutional actors explore during the meetings how they, each using their own position and knowledge, can work together with others towards desired changes in the social policy system.

Secondly, a series of workshops will be co-designed using Techniques of Futuring (ToF) (24). Such techniques have been proven successful in realizing system level changes in other domains, for instance in energy transition (25). ToF's can be defined as 'practices bringing together actors around one or more imagined futures and through which actors come to share particular orientations for action' (25) (p225). More specifically, we achieve this by using various established tools, such as 'whole system in the room', stakeholder consultation, and immersive design. See figure 1 for an overview of the series of workshops.

Stakeholder engagement

Stakeholders involved in the action research are national partners such as professional associations, national government bodies, and interest associations. From the start of the research, they are invited to take an active position in contributing to system change, through their engagement in productive interactive meetings, and reflecting on their own role within the system.

For peer review only - http://bmjopen.bmj.com/site/about/guidelines.xhtml

Data analysis

Qualitative data, such as interviews, field notes and recordings, will be analysed according to thematic content analyses. The analysis focuses on existing and desired institutional dynamics, structures, and logics, mainly expressed through narratives. To ensure quality of analysis, the following measures will be taken into account (26):

- Interpretations will be verified with the institutional partners participating in the action research (member check);

- Multiple data sources will be used and combined in the analyses (data triangulation);

- Data coding will be discussed within the multidisciplinary research team (investigator triangulation);

- Researchers will reflect on their role and influence in the research process (reflexivity);

- Decisions and developments, and the underlying reasons will be documented (audit trail).

Data collection and (continuous) analysis is planned from March 2023 until March 2027. Results can be expected in the course of 2026 and 2027.

Outcomes

Priorities for reducing socioeconomic health inequalities identified in the WRR Policy Brief (5) comprise physical activity and diet, smoking, problematic alcohol use (i.e. lifestyle behaviours) and mental health in groups in vulnerable positions. The selected existing social policy interventions are evaluated on their impact on outcomes related to these priorities. For example: Statistics Netherlands microdata on antidepressant use for mental health (see **supplemental text box 2**).

Target population

For peer review only - http://bmjopen.bmj.com/site/about/guidelines.xhtml

We target two groups with deprivation in (economic) resources: 1) recipients of social assistance benefits (unemployment benefit of last resort), and 2) people with (a risk of) overindebtedness.

Settings

As urban and rural settings might produce differences in vulnerability (i.e. capital) and in governance strategies, having both substantially represented in the study ensures valuable insights for both science and society. In this study, two large municipalities in the strong urbanized West of the Netherlands collaborate. In the more ruralized Mid-East of the Netherlands, 5 municipalities of different sizes (ranging from small to midsize) collaborate.

Patient and public involvement

Citizens participate in the study in two ways. Firstly, a participation board of citizens in vulnerable positions participated in the development of the study protocol. They provided research questions and ideas, and input to decide on the selection (criteria) of potential effective interventions. Moreover, they commented on the raw initial program theory of an intervention in a (realist) focus group interview (please see **supplemental text box 1** for an illustration). Lastly, the participation board will take part in one of the meetings with institutional actors, which focused on the citizens' experiences of the social policy system and its effects on their health.

Secondly, people with mild cognitive limitations are overrepresented among people in vulnerable positions, and so they are an important sub-target group. Therefore, a representative of a national interest group that gives voice to people with cognitive limitations, with lived experiences with overindebtedness and receiving social policy interventions will contribute to the research, in for example participating in the development of (communication) materials for citizens.

ETHICS AND DISSEMINATION

Ethics

For peer review only - http://bmjopen.bmj.com/site/about/guidelines.xhtml

The CCMO, the Dutch Central Commission for Human Research, has assessed this study as not liable to the Medical Research Involving Subjects Act (WMO). This means that it is not required to seek ethical approval by a Medical Ethical Committee. However, advice on ethical aspects related to data collection as discussed below, will be provided by the Social Sciences ethical committee of Wageningen University. Informed consent to participate in the study will be obtained from participants for the use of all forms of personally identifiable data.

It is important that research participants are not harmed or emotionally burdened. However, data collection directly from citizens has a large added value. Their lived experiences provide relevant and accurate information on contexts, mechanisms and outcomes in the realist evaluation of selected existing social policy interventions. Data may be collected about experiences with deprivation in economic resources, social policies and (mental) health. These can be considered sensitive and stigmatized topics, possibly emotionally burdening them and causing psychological distress.

Where possible, less burdensome forms of data collection will be performed, such as using register data and document analyses. However, in case lived experiences provides indispensable insights, risk of emotional burden for participants will be diminished as much as possible, by properly preparing the methods, for example with mock interviews (27). This will help to get familiar with doing interviews about a certain topic, but also prepare for worst-case scenarios. This way, the researcher can learn to deal with for example psychological distress that can arise as a result of the research questions. The questions will also be prepared to be appropriately sensitive and avoid stigmatizing language (27).

Dissemination

The aim of dissemination within RASP is to reach and promote knowledge uptake among institutional actors at multiple system levels, beyond those involved in the study. The national partners involved in the action research have a large constituency among these actors and effective channels for knowledge dissemination and utilization. Dissemination and utilization activities will

For peer review only - http://bmjopen.bmj.com/site/about/guidelines.xhtml

therefore align with these channels. In the beginning of the project, a communication plan will be formulated that maps out activities of actors involved in the study, that may offer opportunities for knowledge dissemination and utilization. The intended target population will have a key role in determining in which form results will be presented (i.e. co-creation).

We identify six types of relevant target groups for knowledge dissemination. These six target groups are listed below, together with tools, materials and activities, considered relevant for them:

1. Citizens with (risk of) problematic debts and/or receiving social assistance benefits

Communication materials will be developed in co-creation with citizens in vulnerable positions, including a representative from the interest group for people with mild cognitive limitations. An example is an icon folder, or videoclip.

2. Students

Educational materials for students of Social Work, and Social and Legal Services will be developed in co-creation with students, lecturers and current professionals, to ensure fitting of the material in the education program and the daily practice, such as an e-learning module or a guest lecture.

3. Professionals working in public services related to social policy

The publications in professional journals that are planned in this project, target mainly the executing professionals.

4. Policymakers and managers in municipal public services related social policy and/or public health

This target group will be reached through different channels and in different forms; factsheets and infographics will be provided to share the direct insights related to the research, contributions will be made to existing communication channels, such as journals, podcasts and blogs, recurrent symposium. In addition, and an end-symposium will be organized.

For peer review only - http://bmjopen.bmj.com/site/about/guidelines.xhtml

5. Policymakers at the national government, working in public health or social policy domain

This target group will be reached throughout the project as representatives of this group actively take part in the action research component, as well as at the end of the project through an (online) symposium.

6. Scientific community

This community will be reached through scientific publications and presentations at relevant conferences, such as the international debt research group that meets annually at the Law & Society Association Conference.

In addition to the specified target populations, the general audience will be informed about milestones in the project by issuing press releases, and/or LinkedIn messages, and/or corporate communication channels.

DISCUSSION

A first strength can be found in the cross-domain systems approach of health inequalities. Many of the various factors influencing health, such as social, economic, environmental, and commercial aspects, which are vital for safeguarding and fostering population health, are in fact not part of the traditional health domain (28). Therefore, the World Health Organization (28) argues to expand the boundaries of what are considered systems that contribute to population health beyond the traditional health systems, into "systems for health". In this perspective, social policy systems are considered to be (potential) systems for health.

A strength of this study protocol is also that it enables both an in-depth understanding of how social policy contributes to health outcomes in citizens in vulnerable positions, and a strategical preparation for uptake and upscaling of these insights. To better understand the mechanisms through which social policy generate health outcomes, and under which contextual circumstances

For peer review only - http://bmjopen.bmj.com/site/about/guidelines.xhtml

these mechanisms can fire, the project conducts a realist evaluation of existing policy interventions embedded in local social policy systems. To strategically prepare for uptake and upscaling of social policy interventions, the project takes an institutional approach to develop such strategies. The combination of these two complementing approaches is both innovative as well as suitable for the required systems change.

Another strength can be found in the settings of this study protocol. Both the forms and causes of vulnerability in populations and social policy practices (including means and possibilities) vary between municipalities, and across rural and urban settings. Both settings are represented in this study by multiple municipalities, varying in size.

A last strength can be found in the transdisciplinarity of this study protocol. To get a full understanding of the workings of social policies, a transdisciplinary team that works in close cooperation is required. The realist approach is particularly appropriate as a catalyst for achieving synergy between disciplines, as it brings together theory in a transdisciplinary way, and methods from different disciplines complement each other. (Behavioural) health sciences is a multidisciplinary field of study, related to health and healthcare. Especially relevant to the project, is expertise from this field of study on (determinants and psychological pathways of) lifestyle behaviours, but also expertise on evaluating complex interventions. Sociology contributes expertise on the study of inequality in access to diverse forms of resources as well as the contextual nature of social phenomena. Empirical micro-economics adds expertise on micro-economic analyses of behaviour of households, with regard to household finance, employment and health outcomes. Governance and organizational science adds expertise on the development of implementation strategies, drawn from institutional approaches. Furthermore, the realist approach can be considered an integrative approach, as stakeholders are involved throughout the research process, including dual theorizing (i.e. integrating scientific theories with stakeholder theories, (29)), and validation of results. In other

For peer review only - http://bmjopen.bmj.com/site/about/guidelines.xhtml

words, in this project, scientific knowledge, is combined with professional knowledge, and experiential knowledge.

Although the cross-domain approach in this study can be considered promising, health inequalities are a genuine so-called wicked problem. Over the past decennia, despite multiple efforts, the gap in life expectancy between the wealthiest, and the least wealthiest group in the Netherlands, have only grown (5). It can be questioned whether social policy intervention can result in sufficient health gains to genuinely reduce health inequality. Possibly, the potential benefit of social policies lies in protecting the current health status of citizens in vulnerable positions (i.e. no further exacerbation of their health status), rather than actually promoting it (i.e. no actual increase in healthy lifestyle behaviour, and improving mental health). However, given the current trend of the ever growing gap, no further exacerbation might then be still considered a relevant step. It might be, that in order to reach health inequality through actual health promotion, not only the social policy domain should be involved, but for example also other policy domains such as the built environment (e.g. the food environment, the physical activity environment, etc.).

Another limitation of the cross-domain approach might be, that some might consider evaluating health impact of policies that have other objectives (such as disposing of sufficient resources) a form of "health imperialism". However, in this study, the primary objectives of social policies (i.e. redistribution of resources) are considered a crucial part of the pathway to health impact. Health is not considered more important than disposing of sufficient resources, but the bi-directional relation of health and disposing of resources is considered crucial for health inequalities. As such, this study protocol aligns with the health *for* all policies- development (29).

Another potential limitation that can be found in reaching the objective of this study, is the challenge that social policy (i.e. how should burdens and resources be distributed in society, and who is deserving of what?) is subject to political ideology. This particularly might play a role in the action research, for example in the willingness to change. Additionally, the political climate itself

For peer review only - http://bmjopen.bmj.com/site/about/guidelines.xhtml

might change, which, through institutional design, might lead to a challenge for support among stakeholders.

Funding

This work is supported by the Dutch Research Agenda, executed by NWO and ZonMw, grant number NWA 1333.19.001. Independent peer reviews were part of the application process.

Contributors

J.v.B. led the development of the study protocol, and is the research guarantor. J.v.B., E.J.d.B., M.W., Y.M.l.G., E.v.B., H.v.d.H., S.T., M.H., A.H., T.M., M.K., E.d.V. have provided intellectual input and critically reviewed the study protocol. All authors approved the final manuscript.

Competing interests

The authors declare that they have no competing interests.

Acknowledgements

The authors thank the citizens in the advisory board "a healthy future for everyone", as well as the policy advisors of the collaborating municipalities for their involvement and contributions to the development of this study protocol.

Consent for publication

Not applicable.

For peer review only

For peer review only - http://bmjopen.bmj.com/site/about/guidelines.xhtml

References

1. Vrooman, C., Gijsberts, M., Boelhouwer, J., & Mantext, M. Verschil in Nederland. 2014. (SCP-publicatie).

2. RVS. Gezondheidsverschillen voorbij; Complexe ongelijkheid is een zaak van ons allemaal [Internet]. 2020. Available from: https://adviezen.raadrvs.nl/gezondheidsverschillen-voorbij/

3. Pinxten W, Lievens J. The importance of economic, social and cultural capital in understanding health inequalities: using a Bourdieu-based approach in research on physical and mental health perceptions. Sociol Health Illn. 2014 Sep;36(7):1095–110.

4. Veenstra G, Abel T. Capital interplays and social inequalities in health. Scand J Public Health. 2019 Aug;47(6):631–4.

5. Broeders, D.W.J., Das, H.D., Jennissen, R.P.W., Tiemeijer W.L. & de Visser, M. Van verschil naar potentieel: een realistisch perspectief op de sociaaleconomische gezondheidsverschillen [Internet]. Vol. WRR Policy Brief. 2018. Available from: https://www.wrr.nl/publicaties/policy-briefs/2018/08/27/van-verschil-naar-potentieel.-een-realistisch-perspectief-op-de-sociaaleconomische-gezondheidsverschillen

6. Statistics Netherlands. Personen met een uitkering; uitkeringsontvangers per uitkeringssoort [Internet]. 2021. Available from: https://www.cbs.nl/nl-nl/cijfers/detail/83426NED?q=bijstand

7. Rijksoverheid. Factsheet Participatiewet [Internet]. Ministerie van Sociale Zaken en Werkgelegenheid; 2019. Available from: https://www.rijksoverheid.nl/documenten/brochures/2019/11/26/participatiewet

8. Van Rijnsoever MP, Tromp E, Waterlander WE, Schütz FN, Steenhuis IHM. Verschillen in leefstijl en gezondheid tussen mensen met en zonder schulden. Tijdschr Voor Gezondheidswetenschappen. 2011 Jan;89(1):43–50.

9. Yildiz B, Schuring M, Knoef MG, Burdorf A. Chronic diseases and multimorbidity among unemployed and employed persons in the Netherlands: a register-based cross-sectional study. BMJ Open. 2020 Jul;10(7):e035037.

10. Einerhand, M., & Ravesteijn, B. Psychische klachten en de arbeidsmarkt. 102(4754):481–3.

11. Cartwright N. Are RCTs the Gold Standard? BioSocieties. 2007 Mar;2(1):11–20.

12. Craig P, Dieppe P, Macintyre S, Michie S, Nazareth I, Petticrew M. Developing and evaluating complex interventions: the new Medical Research Council guidance. BMJ. 2008 Sep 29;a1655.

13. Craig P, Dieppe P, Macintyre S, Michie S, Nazareth I, Petticrew M. Developing and evaluating complex interventions: The new Medical Research Council guidance. Int J Nurs Stud. 2013 May;50(5):587–92.

14. Larouche A, Potvin L. Stimulating innovative research in health promotion. Glob Health Promot. 2013 Jun;20(2):64–9.

For peer review only - http://bmjopen.bmj.com/site/about/guidelines.xhtml

15. Wong G, Westhorp G, Manzano A, Greenhalgh J, Jagosh J, Greenhalgh T. RAMESES II reporting standards for realist evaluations. BMC Med. 2016 Dec;14(1):96.

16. Pawson, R., & Tilley, N. Realistic evaluation. SAGE Publications Ltd; 1997.

17. Westhorp G. Understanding Mechanisms in Realist Evaluation and Research. In: Doing Realist Research [Internet]. 1 Oliver's Yard, 55 City Road London EC1Y 1SP: SAGE Publications Ltd; 2018 [cited 2023 Oct 2]. p. 41–58. Available from: https://methods.sagepub.com/book/doing-realist-research/i249.xml

18. Salter KL, Kothari A. Using realist evaluation to open the black box of knowledge translation: a state-of-the-art review. Implement Sci. 2014 Dec;9(1):115.

19. Emmel N, Greenhalgh J, Manzano A, Monaghan M, Dalkin S. Doing Realist Research [Internet]. 1 Oliver's Yard, 55 City Road London EC1Y 1SP: SAGE Publications Ltd; 2018 [cited 2023 Oct 2]. Available from: https://methods.sagepub.com/book/doing-realist-research

20. Jagosh J. Retroductive theorizing in Pawson and Tilley's applied scientific realism. J Crit Realism. 2020 Mar 14;19(2):121–30.

21. Greenwood R, Oliver C, Lawrence T, Meyer R. The SAGE Handbook of Organizational Institutionalism [Internet]. 1 Oliver's Yard, 55 City Road London EC1Y 1SP: SAGE Publications Ltd; 2017 [cited 2023 Oct 2]. Available from: https://sk.sagepub.com/reference/sage-handbook-of-organizational-institutionalism-2e

22. Lopes AV, Farias JS. How can governance support collaborative innovation in the public sector? A systematic review of the literature. Int Rev Adm Sci. 2022 Mar;88(1):114–30.

23. Burns D. Systemic Action Research: A Strategy for Whole System Change [Internet]. 1st ed. Bristol University Press; 2007 [cited 2023 Oct 2]. Available from: https://www.cambridge.org/core/product/identifier/9781847422750/type/book

24. Oomen J, Hoffman J, Hajer MA. Techniques of futuring: On how imagined futures become socially performative. Eur J Soc Theory. 2022 May;25(2):252–70.

25. Hajer MA, Pelzer P. 2050—An Energetic Odyssey: Understanding 'Techniques of Futuring' in the transition towards renewable energy. Energy Res Soc Sci. 2018 Oct;44:222–31.

26. Frambach JM, van der Vleuten CP, Durning SJ. AM Last Page: Quality Criteria in Qualitative and Quantitative Research. 2013;88(4):552.

27. Melville, A. H D. Conducting Sensitive Interviews: A Review of Reflections. Law Method. 2016. 10.5553/REM/.000015

28. World Health Organization, Shroff ZC, Marten R, Hanson K. Systems for health: everyone has a role: flagship report of the Alliance for Health Policy and Systems Research [Internet]. Geneva: World Health Organization; 2022. Available from: https://iris.who.int/handle/10665/363923

29. Greer SL, Falkenbach M, Siciliani L, McKee M, Wismar M, Figueras J. From Health in All Policies to Health for All Policies. Lancet Public Health. 2022 Aug;7(8):e718–20.

For peer review only - http://bmjopen.bmj.com/site/about/guidelines.xhtml

FIGURE TITLE

Figure 1. Overview of the co-designed workshops, to develop anticipatory governance strategies from an institutional approach

For peer review only

For peer review only - http://bmjopen.bmj.com/site/about/guidelines.xhtml

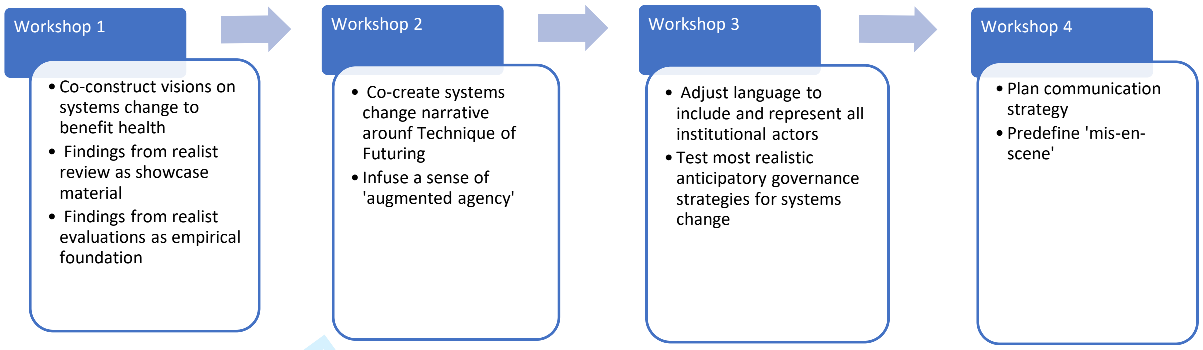

For peer review only

Supplemental File 1 STATE OF THE ART: SOCIAL POLICY AND HEALTH

In this section, we summarize the state of the art of research on the effects of social policy on health. In general, the underlying mechanisms and transferability of findings to a Dutch-type welfare context often remains unclear. Looking at the Netherlands, there are even substantial differences between regions (urban and rural) and municipalities. In line with the two main target groups for this project, namely people who receive social assistance and people facing problematic debt, we summarize the state of the art of the health impact of two types of social policy that they are subject to, namely social benefits policy, and debt policy. We tailor our summary towards dimensions of social policies that can be modified in social policy interventions and that point towards opportunities for improved realization of health potential. Strikingly, there is much more known about social assistance policy than debt policy. Research on the Netherlands is scarce in general and there is little to no research that takes an explicit complex systems approach.

Impact of Social Assistance Benefits on Health Outcomes

The literature distinguishes three relevant dimensions of social benefit policies that might affect health outcomes: 1) generosity and eligibility, 2) active labour market policies, and 3) requirements, monitoring and sanctions. Considering 1), in the Netherlands, social assistance is a non-contributory, monthly transfer scheme of last resort for unemployed people who do not or no longer qualify for other benefit schemes, including unemployment benefits. Eligibility for this scheme is based on a work- and means-test, while the benefit level depends on the household composition. In view of 2) labour and reintegration requirements in the Netherlands including active search for a job, willingness to accept all types of paid employment offered, participation in active labour market programs, following education and skill-development programs, signing up with a temporary employment agency (if requested), and performing volunteer work. Lastly 3), as is common to such schemes (1), social assistance claimants have to comply with regulations and obligations, such as

For peer review only

administrative requirements (e.g., providing information, attending appointments, and responding to letters and emails) to maintain (full) entitlement to the benefit.

1. Effects of generosity and eligibility of social benefit systems

Findings of a recent literature review show that widened eligibility and increased generosity of social benefit systems are associated with improved mental health and reduced mental health inequalities (2). Similarly, reduced eligibility and generosity were associated with deteriorated mental health and increased mental health inequalities.

These latter findings were in line with findings of a literature review examining the health effect of social assistance programs (3). This review shows that stricter eligibility and generosity result in worse health outcomes. Simpson et al. (2) distinguish mechanisms at two levels that might underly these effects: 1) at the aggregate level (e.g., lower generosity and stricter eligibility criteria coincide with stigmatizing attitudes toward benefit claimants and increased income inequality which may subsequently impair mental health) and 2) at the individual level (e.g., increased generosity and eligibility may hamper employment prospects of claimants which subsequently impair mental health). The direction of the effects of expansionary and contractionary policies on mental health is not straightforward as these policies might trigger mechanisms both improving and harming mental health. The underlying mechanisms are complex and might depend on moderating factors.

However, widened eligibility may not improve health in all contexts. In the Netherlands, a randomized experiment among social assistance claimants in The Netherlands shows that imposing a 1-month job search period upon applicants lowered welfare take-up and increased employment rates, while this stricter eligibility regulation did not affect (mental) health (4).

2. Effects of active labor market policies (ALMPs)

For peer review only

A second aspect of social benefits policy that is relevant for health outcomes are Active Labor Market Policies (ALMPs). ALMPs aim to increase re-employment rates and include job search assistance, job or vocational training, and subsidized public and private employment. In general, literature reviews and meta-studies have found that ALMPs have small effects on re-employment in the short run, but larger effects in the medium and long run (e.g., (5,6). Job search assistance programs focusing on 'work first' have similar effects in the short and long run, while job training and private sector employment programs increase employment only in the medium and longer run (probably due to lock in effects).

A literature review about the effects of ALMPs on self-reported health outcomes showed that job search assistance including a psychological component (e.g., enhancing self-efficacy) have positive effects on mental health (7). In general, high-risk groups benefitted most. Effects of vocational training programs and subsidized employment were mostly positive but reflected more diversity. In a recent study using administrative data, Caliendo et al. (8) found that participation in a vocational training program reduced drug prescriptions for cardiovascular and mental health problems by about 6-8 percent, while sickness absence reduced by about 20 percent. This effect was larger for vulnerable groups (e.g., lower educated claimants). Effects were likely to be direct (via adoption of a daily routine) rather than indirect (via improved employment prospects) as the reduction in drugs prescription started when claimants were still enrolled in the training program.

Findings in the Netherlands are generally consistent with international evidence. As found in the Dutch social assistance context, entering paid employment has a positive impact on mental and physical health (9). As a consequence, active labor market policies might act as important health promotion policies among unemployed individuals. In the Dutch social assistance context, welfare departments usually provide employment services (including job search assistance and regular encounters with caseworkers) following a target-group based approach. Schuring et al. (10) investigated the effects of different types of employment service approaches for Dutch social

For peer review only

assistance claimant using a quasi-experimental approach. They found that Matching (approach focusing on facilitating fast re-employment) reduced drug prescriptions for mental health problems by six percentage points compared to Pre-matching (focus on reducing labor market barriers). These approaches were not significantly different in their impact on other health outcomes (cardiovascular diseases, diabetes, and respiratory illness). Matching also resulted in higher re-employment rates than Pre-matching which might explain the positive finding on mental health. These findings suggest that ALMPs could be important to realize a structural breakthrough in improving mental health among vulnerable individuals.

3. Effects of requirements, monitoring, and sanction policy

A third aspect of social benefits policy that can affect health outcomes is the enforcement regime. Economic studies have shown that stricter labor and reintegration requirements, increased monitoring, and more stringent sanction policy tend to decrease unemployment duration and increase job entry rates of (sanctioned) claimants (11–14), although effects often disappear in the long run and these policies might reduce post-unemployment job quality (15–17).

Only a few studies investigated the impact of (elements off) the enforcement regime on health outcomes. Some qualitative and correlational studies conducted in the UK suggest that stricter labour and reintegration requirements, monitoring, and sanction policy are predominantly experienced as stressful and punitive and may negatively affect the mental health and well-being of claimants (18–20). Among claimants of a Danish unemployment benefit program, Baekgaard et al. (21) found that reducing compliance demands to labour and reintegration requirements lowered reported stress levels and increased autonomy. They did not find an effect on stigma. Caliendo et al. (8) investigated the effects of imposed benefit sanctions due to non-compliance to labour and reintegration requirements. They found an increase in drug prescriptions for mental health problems in the month before sanction was imposed (probably due to receiving a warning) and an increase in sickness absence in the months after the sanction was imposed. They did not find a long-run effect

For peer review only

on drug prescriptions related to cardiovascular diseases and mental health problems. A potential explanation is that negative health effects of being sanctioned due to financial stress might be compensated by a positive health effect via increased re-employment rates. Understanding the mechanisms in the Dutch context would help to design effective policies with positive health effects both in the short and the long run.

Impact of Debt Policy on Health Outcomes

In comparison with studies on social assistance benefits policies, studies investigating the effects of debt policy or interventions on health are scarce. We can distinguish three dimensions of debt policy for potential health outcomes: 1) debt counselling, 2) altering the composition of creditors, and 3) debt restructuring and reduction. In the Netherlands, those dimensions are highly interrelated in debt policy. People who are unable to repay their debts on their own can apply for voluntary or statutory debt restructuring based on the law *Wet schuldsanering natuurlijke personen* (Wsnp) (translated into Natural Persons Debt Rehabilitation Act). Municipalities appoint debt counsellors who develop a voluntary payment arrangement based on a calculation of how much the debtor can pay to the creditors each month over a period of three years. If the creditors accept the voluntary payment plan, and the debtor adheres to the plan without entering new debts, the remaining debt is acquitted after three years. If the creditors fail to agree to the payment plan, the citizen can apply for statutory debt restructuring. If the application is granted, the judge appoints an administrator who oversees the process and creditors are obliged to agree with the payment plan. Similar to a voluntary plan, after three years of payments, remaining debts are absolved. Despite this apparent protection offered to people with problematic debts under the law, only a fraction (ca. 14%) of eligible individuals actually seek assistance (22). To make matters worse, the stabilization process that precedes the voluntary repayment plan often takes months and has a high dropout rate. In addition, municipal approaches are often not well tailored to the complex realities of the lives of

For peer review only

people with problematic debts, and creditors are not always cooperative. Municipalities differ in the conditions and requirements they impose. For example 20% of the municipalities ask for documentation of the financial situation prior to enrolment, which can be a significant burden for people in problematic debt. It is safe to state that debt policy now fails to reach the large majority of this vulnerable target group, though there are mayor differences between municipalities.

1. Debt counselling

Of the three dimensions, most evidence is available on the effect of debt counselling on health. A cross-sectional study found that debt counselling significantly reduced financial stressor events among its clients, which in turn was associated with a more positive perception of one's financial situation and perceived health. There were no significant direct effects of debt counselling on perceived health, possible due to the follow-up period of 18 months being too short (23). Similarly, a literature review on the effects of financial counselling shows that there is little evidence for the effect of financial counselling on health. This may be explained by the characteristics of interventions. Most of the interventions that were evaluated put great emphasis on knowledge, while applying behavioural insights might increase the effectiveness of counselling (24). One example of an intervention used in debt counselling that incorporates several behavioural insights is Mobility Mentoring, which was developed by the US-based organization EMPath. Mobility Mentoring has shown promising initial results in the US, but has yet to be proven effective (25). There is scarce evidence in the Netherlands on the effect of debt counselling on client health. A process evaluation of Mobility Mentoring at a Dutch municipality showed that after six months of assistance, clients reported that their health problems were less hindering for their participation in caretaker, educational or work-related activities. One study compared three types of debt counselling In the Netherlands (26). Intensive support across multiple life domains for clients with complex problems, but also target financial counselling combined with administrative and legal support for relatively self-sufficient clients were both suggested to be successful. Success in

For peer review only

addressing financial problems coincided with less stress and more belief in a positive outcome in both groups. A counselling type with more limited support accessible for all client types generated less success and less positive health outcomes. Clients experience myriad of emotions, for which there is little attention in the counselling sessions(26). The authors believe that more attention for the client's experience may reduce the attrition rate of debt restructuring trajectories.

2. Altering the composition of creditors

There is evidence that a smaller number of creditors might have positive health effects. A quasi-experimental study compared households in Singapore that received a certain amount of money to pay off their debts (27). All households received the same amount, which translated into different numbers of creditors that were paid off. Each additional creditor that was paid off was associated with an improved cognitive functioning by a quarter of standard deviation, and 11% less anxiety and 10% less present bias. A qualitative study among UK citizens showed that borrowing from microcredit providers was perceived as having positive effects on health (28). Micro credit reduced stress in the face of unexpected financial costs; it enabled them to maintain social relationships and increased the perceived control over their lives.

3. Debt restructuring and reduction

In a qualitative study on re-employment among participants of debt restructuring programs, participants reported that the start of the voluntary payment plan led to less mental and physical health complaints, such as stress, sleeping problems, and feelings of depression (29).

GAPS IN KNOWLEDGE

While there are some promising leads for reducing health inequalities through social policy, there is still much to be clarified. As discussed, studies investigating the health effects of debt policies are

For peer review only

scarce. The literature reviews on social assistance benefit policies report or show a lack of studies conducted in a Dutch-type welfare context.

In addition, there is a lack of studies unravelling the mechanisms underlying the effects of social policies on health outcomes. As a consequence of these gaps, we should be cautious in generalizing the results to the Dutch social assistance benefits context. The present research project aims to fill these gaps for both policy domains. In general, health impacts are usually an unintended by-product of policies that are themselves complex and such effects are embedded and possibly counteracted in a complex system (30). In addition, studies usually fail to account for contextual differences (e.g., rural and urban settings) and to address both ex-ante and ex-post effects of social policies. An approach is necessary that combines evidence on impacts, but that also accounts for the way in which contextual variables interact with policy.

REFERENCES

1. Immervoll,H. K C. How demanding are activation requirements for jobseekers [Internet]. 2018 Jul [cited 2023 Oct 2]. (OECD Social, Employment and Migration Working Papers; vol. 215). Report No.: 215. Available from: https://www.oecd-ilibrary.org/employment/how-demanding-are-activation-requirements-for-jobseekers_2bdfecca-en
2. Simpson J, Albani V, Bell Z, Bambra C, Brown H. Effects of social security policy reforms on mental health and inequalities: A systematic review of observational studies in high-income countries. Soc Sci Med. 2021 Mar;272:113717.
3. Shahidi FV, Ramraj C, Sod-Erdene O, Hildebrand V, Siddiqi A. The impact of social assistance programs on population health: a systematic review of research in high-income countries. BMC Public Health. 2019 Dec;19(1):2.
4. Bolhaar J, Ketel N, Van Der Klaauw B. Job Search Periods for Welfare Applicants: Evidence from a Randomized Experiment. Am Econ J Appl Econ. 2019 Jan 1;11(1):92–125.
5. Card D, Kluve J, Weber A. What Works? A Meta Analysis of Recent Active Labor Market Program Evaluations. J Eur Econ Assoc. 2018 Jun 1;16(3):894–931.
6. Vooren M, Haelermans C, Groot W, Maassen Van Den Brink H. THE EFFECTIVENESS OF ACTIVE LABOR MARKET POLICIES: A META-ANALYSIS. J Econ Surv. 2019 Feb;33(1):125–49.
7. Puig-Barrachina V, Giró P, Artazcoz L, Bartoll X, Cortés-Franch I, Fernández A, et al. The impact of Active Labour Market Policies on health outcomes: a Scoping review. Eur J Public Health [Internet]. 2019 Mar 25 [cited 2023 Oct 2]; Available from: https://academic.oup.com/eurpub/advance-article/doi/10.1093/eurpub/ckz026/5419277
8. Caliendo M, Mahlstedt R, Van Den Berg GJ, Vikström J. Side Effects of Labor Market Policies. SSRN Electron J [Internet]. 2020 [cited 2023 Oct 2]; Available from: https://www.ssrn.com/abstract=3730444
9. Schuring M, Robroek SJ, Burdorf A. The benefits of paid employment among persons with common mental health problems: evidence for the selection and causation mechanism.

Scand J Work Environ Health [Internet]. 2017 Oct 2 [cited 2023 Oct 2]; Available from: http://www.sjweh.fi/show_abstract.php?abstract_id=3675

10. Schuring M, Knoef M, Burdorf A. The effect of employment services on employment and health among unemployed persons: an IV approach. Eur J Public Health. 2022 Oct 21;32(Supplement_3):ckac131.263.
11. Abbring JH, Berg GJ, Ours JC. The Effect of Unemployment Insurance Sanctions on the Transition Rate from Unemployment to Employment. Econ J. 2005 Jul;115(505):602–30.
12. Arni P, Schiprowski A. Job search requirements, effort provision and labor market outcomes. J Public Econ. 2019 Jan;169:65–88.
13. Svarer M. The Effect of Sanctions on Exit from Unemployment: Evidence from Denmark: Effect of Sanctions on Exit from Unemployment. Economica. 2011 Oct;78(312):751–78.
14. Van Den Berg GJ, Van Der Klaauw B, Van Ours JC. Punitive Sanctions and the Transition Rate from Welfare to Work. J Labor Econ. 2004 Jan;22(1):211–41.
15. Arni P, Lalive R, Van Ours JC. HOW EFFECTIVE ARE UNEMPLOYMENT BENEFIT SANCTIONS? LOOKING BEYOND UNEMPLOYMENT EXIT: BENEFIT SANCTIONS BEYOND UNEMPLOYMENT EXIT. J Appl Econom. 2013 Nov;28(7):1153–78.
16. Lachowska M, Meral M, Woodbury SA. Effects of the unemployment insurance work test on long-term employment outcomes. Labour Econ. 2016 Aug;41:246–65.
17. Van Den Berg GJ, Vikström J. Monitoring Job Offer Decisions, Punishments, Exit to Work, and Job Quality. Scand J Econ. 2014 Apr;116(2):284–334.
18. Dwyer P, Scullion L, Jones K, McNeill J, Stewart ABR. Work, welfare, and wellbeing: The impacts of welfare conditionality on people with mental health impairments in the UK. Soc Policy Adm. 2020 Mar;54(2):311–26.
19. Wright S, Fletcher DR, Stewart ABR. Punitive benefit sanctions, welfare conditionality, and the social abuse of unemployed people in Britain: Transforming claimants into offenders? Soc Policy Adm. 2020 Mar;54(2):278–94.
20. Wright S, Patrick R. Welfare Conditionality in Lived Experience: Aggregating Qualitative Longitudinal Research. Soc Policy Soc. 2019 Oct;18(4):597–613.
21. Baekgaard M, Mikkelsen KS, Madsen JK, Christensen J. Reducing Compliance Demands in Government Benefit Programs Improves the Psychological Well-Being of Target Group Members. J Public Adm Res Theory. 2021 Sep 13;31(4):806–21.
22. Schonewille, G. & Weijers, M. Veel schuldenaren weten schuldhulp slecht te vinden. ESB. 2019;104(4776).
23. Kim, Jinhee and Garman, E. Thomas and Sorhaindo, Benoit,. Relationships Among Credit Counseling Clients' Financial Wellbeing, Financial Behaviors, Financial Stressor Events, and Health [Internet]. Journal of Financial Counseling and Planning; 2003. Available from: https://ssrn.com/abstract=2265623
24. Jungmann, N., Madern, T. Duurzame verandering van financieel gezond gedrag: Droom of werkelijkheid? [Internet]. WRR; 2016. Available from: https://www.wrr.nl/publicaties/working-papers/2016/06/30/duurzame-verbetering-van-gezond-financieel-gedrag.-droom-of-werkelijkheid#:~:text=Droom%20of%20werkelijkheid%3F%20is%20uitgevoerd,gezonder%20financieel%20gedrag%20gaan%20vertonen.
25. Washington State Department of Early Learning. Year one report - ECEAP Family Support Pilot june 2015 -june 2016 [Internet]. Olympia; 2016. Available from: https://dcyf.wa.gov/sites/default/files/pdf/eceap/ECEAP_Family_Support_Pilot_Report_Final.pdf

For peer review only

26. van Logtestijn, B. van der Want,E. van Jeveren, F., Dirven, J. & Broere, M. Gemeenten zetten de schouders eronder [Internet]. 2019. Available from: https://vng.nl/publicaties/gemeenten-zetten-de-schouders-eronder
27. Ong Q, Theseira W, Ng IYH. Reducing debt improves psychological functioning and changes decision-making in the poor. Proc Natl Acad Sci. 2019 Apr 9;116(15):7244–9.
28. Myrskylä M, Gagnon A, Bengtsson T. Pathways to Health and Well-being. Soc Sci Med. 2014 Oct;119:175–9.
29. van der Laan, J. & van Geuns, R. Schuldenvrij: De weg naar werk? [Internet]. 2016. Available from: https://www.hva.nl/kmr/gedeelde-content/publicaties/publicaties-algemeen/2016/schuldenvrij-de-weg-naar-werk.html
30. Shiell A, Hawe P, Gold L. Complex interventions or complex systems? Implications for health economic evaluation. BMJ. 2008 Jun 7;336(7656):1281–3.

For peer review only

Supplemental Text box 1 ILLUSTRATION OF A SOCIAL POLICY INTERVENTION AND ITS INITIAL

"Vroeg Eropaf" (Early Outreach) is a debt policy intervention designed to address starting problematic debts through a multi-component approach. This illustration provides an overview of the intervention, presents the initial program theory, and outlines the proposed research methodology for testing and refining the theory.

The Vroeg Eropaf intervention aims to identify and address starting problematic debts through three key components. Firstly, a network of service providers signals arrears in payment to municipalities when individuals experience financial difficulties. Secondly, social service teams from municipalities make contact with households when at least two service providers signal arrears, offering assistance if needed. Lastly, if assistance is desired, the teams provide support in managing financial problems by assigning individuals to debt service departments or other societal organizations. This intervention adheres to Dutch and European privacy regulations, and Dutch municipalities have a legal duty to engage in early signaling of debt since January 1st, 2021. Vroeg Eropaf is currently implemented in multiple municipalities, both in urban and rural settings, and has been identified as a potentially relevant intervention for this research.

The initial program theory is informed by existing literature on the impact of overindebtedness on health outcomes. Studies have shown a correlation between debt and various mental health issues, including mental disorders, depression, suicide attempts, problem drinking, drug dependence, neurotic disorders, and psychotic disorders. The underlying mechanisms linking overindebtedness to poor health involve worry, financial scarcity, stress, negative affect, social isolation, reduced perceived control, reduced sleep quality, failure to seek assistance, poor medication adherence, impaired executive functioning, and unhealthy lifestyle behaviors such as unhealthy eating, smoking, and reduced physical activity.

Furthermore, the theory proposes that the reduction of administrative burdens, which individuals may experience in their interactions with the state, plays a significant role in improving health outcomes. Administrative burdens consist of learning costs (searching for information about social programs), compliance costs (meeting administrative requirements), and psychological costs (stress, frustration, loss of autonomy, and stigma). The assistance component of Vroeg Eropaf aims to alleviate these burdens, potentially reducing barriers to accessing debt services and improving overall well-being.

However, it is important to note that the intervention may not have a positive impact on health outcomes for certain groups or under specific conditions. Challenges may arise in effectively reaching all citizens with starting problematic debts, especially if the network of service providers fails to adequately signal or if there are capacity limitations or barriers related to language, literacy, or cultural differences. Additionally, behavioral changes, such as improved financial behavior or increased uptake of debt services, may not occur for some approached individuals due to unproductive interactions with professionals or a lack of trust.

Stakeholder involvement is a crucial aspect of this research, employing 'dual theorizing' by consulting policy advisors and citizens in vulnerable positions. Stakeholders recognize the potential relevance of Vroeg Eropaf for improving health outcomes. Citizens highlighted the interaction between mental health and their response to the intervention, emphasizing that poor mental health can hinder help-seeking behavior. Other stakeholders also acknowledged the significance of administrative burden, considering it a sort of "hygiene condition" that, when present, exacerbates individuals' situations without guaranteeing improvement upon its reduction. Policy advisors expressed an awareness of the burdens placed on citizens and sought alternatives to alleviate them. In a rural municipality, the concept of 'noaberschap' (neighborship), a form of social resource rooted in historical tradition, was identified as potentially impacting the intervention's outcomes by either triggering feelings of shame and hampering health outcomes, or strengthening social resource and improving health outcomes.

PROGRAM THEORY

For peer review only - http://bmjopen.bmj.com/site/about/guidelines.xhtml

Supplemental Text box 2 ILLUSTRATION OF A MULTI-METHOD EVALUATION STRATEGY

A mixed-method evaluation strategy is utilized to explore various dimensions and levels within the program theory. A combination of document analyses, quasi-experimental design, realist interviews and ethnographic institutional analysis is under consideration for the evaluation of "Vroeg Eropaf" (Early Outreach, see text box 1).

Document analysis entails a meticulous examination of the intervention's implementation and its reach in the municipality under study. This analytical process serves as the foundation for understanding relevant micro, meso, and macro levels and dimensions to be investigated.

We could estimate effects of the Vroeg Eropaf-intervention on resource-related (e.g., labor status and debt situation) and health-related (mental health, physical health) outcomes. To gain insight in the heterogeneity of intervention effects, we will additionally examine whether intervention effects differ among subgroups (e.g., high-risk groups related to (mental) health problems). To identify these intervention effects, we **link administrative data of municipalities to register data of Statistics Netherlands**. Register data of Statistics Netherlands provide individual- and household-level data for all Dutch residents. The data covers several topics ranging from demographics, education, and labour market status to prescribed medication and problematic debts.

Linking municipality data to this data register of Statistics Netherlands provide a unique opportunity to investigate the outcomes of (local) social policy interventions. Recent studies have shown that linking these data sources is feasible and appropriate to identify intervention outcomes of social policy interventions (e.g., 19, 20). To estimate intervention outcomes, we can use quasi-experimental methods, such as instrumental variable approaches, difference-in-difference analyses, regression discontinuity designs, and propensity score matching. These methods fit realist evaluation, as it allows to test the transfactuality of Context-Mechanism-Outcome configurations (60). An appropriate method will be chosen depending on the context characteristics and the availability of data.

Furthermore, on a microlevel, we can investigate the workings of the aforementioned potential mechanisms such as administrative burden, and the contexts that trigger (or prevents them) the mechanisms, such noaberschap in a rural municipality, through **(realist) interviews** with citizens.

Ethnographic institutional analysis of the collaboration between institutional actors allows to explore how micro patterns of professional behavior play a role in the intervention on a mesolevel, and to explore systems-design issues on a macro-level.

Ultimately, this comprehensive combination of methods is aimed at refining, refuting, or revising the context-mechanism-outcome configurations within the program theory, underpinning the workings of the social policy intervention.
