# Supplementary material for: Realist Approach to Social Policies (RASP) study to reduce socioeconomic health inequalities through systems change: protocol for a research project combining mixed-methods realist research with institutional action research
Source: BMJ Open. 2024 Jun 13;14(6):e088571. doi: 10.1136/bmjopen-2024-088571 (PMC11177664; doi:10.1136/bmjopen-2024-088571)
Supplement: Reviewer comments [file bmjopen-2024-088571.reviewer_comments.pdf]

PEER REVIEW HISTORY

BMJ Open publishes all reviews undertaken for accepted manuscripts. Reviewers are asked to complete a checklist review form (**http://bmjopen.bmj.com/site/about/resources/checklist.pdf**) and are provided with free text boxes to elaborate on their assessment. These free text comments are reproduced below.

ARTICLE DETAILS

**TITLE (PROVISIONAL)**	The Realist Approach to Social Policies (RASP) study to reduce socioeconomic health inequalities through systems change: protocol for a research project combining mixed-methods realist research with institutional action research
**AUTHORS**	Van Berkel, Jantien; de Bruijn, Ernst-Jan; Waardenburg, Maikel; la Grouw, Yvonne; van Bennekom, Eline; van der Horst, Hilje; Tonnon, Susanne; Haggenburg- Mohammed, Milou; Haveman-Nies, Annemien; Madern, Tamara; Knoef, Marike; de Vet, Emely

This article was not externally reviewed at BMJ Open. Protocols that have been independently assessed prior to submission to BMJ Open are usually fast-tracked to publication on the grounds that further substantial changes will not be possible. This independent assessment will usually be external, independent review for both a substantial grant award from a non-commercial or government funder and ethics approval.
